# miRNA Profiling and Its Role in Multi-Omics Regulatory Networks Connected with Somaclonal Variation in Cucumber (*Cucumis sativus* L.)

**DOI:** 10.3390/ijms23084317

**Published:** 2022-04-13

**Authors:** Magdalena Ewa Pawełkowicz, Agnieszka Skarzyńska, Marek Daniel Koter, Szymon Turek, Wojciech Pląder

**Affiliations:** Department of Plant Genetics, Breeding and Biotechnology, Institute of Biology, Warsaw University of Life Sciences, 02-776 Warsaw, Poland; agnieszka_skarzynska@sggw.edu.pl (A.S.); marek_koter@sggw.edu.pl (M.D.K.); szymon_turek@sggw.edu.pl (S.T.); wojciech_plader@sggw.edu.pl (W.P.)

**Keywords:** miRNA, sRNA sequencing, sRNA-seq, in vitro culture, cucumber somaclonal variation, regulatory mechanism

## Abstract

The role of miRNAs in connection with the phenomenon of somaclonal variation, which occurs during plant in vitro culture, remains uncertain. This study aims to investigate the possible role of miRNAs in multi-omics regulatory pathways in cucumber somaclonal lines. For this purpose, we performed sRNA sequencing (sRNA-seq) from cucumber fruit samples identified 8, 10 and 44 miRNAs that are differentially expressed between somaclones (S1, S2, S3 lines) and the reference B10 line of *Cucumis sativus.* For miRNA identification, we use ShortStack software designed to filter miRNAs from sRNAs according to specific program criteria. The identification of predicted in-silico targets revealed 2,886 mRNAs encoded by 644 genes. The functional annotation of miRNA’s target genes and gene ontology classification revealed their association with metabolic processes, response to stress, multicellular organism development, biosynthetic process and catalytic activity. We checked with bioinformatic analyses for possible interactions at the level of target proteins, differentially expressed genes (DEGs) and genes affected by genomic polymorphisms. We assume that miRNAs can indirectly influence molecular networks and play a role in many different regulatory pathways, leading to somaclonal variation. This regulation is supposed to occur through the process of the target gene cleavage or translation inhibition, which in turn affects the proteome, as we have shown in the example of molecular networks. This is a new approach combining levels from DNA-seq through mRNA-seq, sRNA-seq and in silico PPI in the area of plants’ somaclonal variation.

## 1. Introduction

For over forty years, it has been reported that the genetic variation in regenerated plants can be induced de novo as a result of the tissue culture environment [1]. However, earlier work indicates that in vitro tissue cultures are susceptible to mutations [2], and the occurrence of many phenotypic changes were later described [3,4]. This variability was defined as somaclonal variation and is defined as the genetic variation observed among the progeny of plants regenerated from somatic cells cultured in vitro [1]. Acquiring plants through tissue cultures is associated with the appearance of changes of a different nature. One of them is genetic variation, manifested by the presence of an increased frequency of point mutations [5], chromosome instability, structural changes and aneuploidy [6,7] and the activation of mobile elements [8]. Moreover, the coexistence of genetic and epigenetic changes resulting from in vitro culture was detected in regenerated crops, e.g., in rye [9], barley [10] and wheat [11]. Somaclonal variation is thought to be a negative phenomenon, for example introducing deleterious traits to the resulting plants. It is still not possible to predict the results of in vitro regeneration. It is random, and in many cases, there is no repeatability, and a large number of genetic changes are based on point mutations or chromosome rearrangements that occur in the segregation of the R1 generation [12,13]. On the other hand, somaclonal variation in in vitro regeneration can be a positive force in inducing a new variety and a source of useful new traits, while being cheaper than conventional breeding. Many agronomic traits that resulted from somaclonal variation have been exploited in crop breeding [9,14]. The phenomenon of somaclonal variation is being investigated, and new aspects of analysis are emerging, such as the role of miRNA in this process. Until now, miRNAs have been studied more in the context of in vitro cultures rather than the phenomenon of variability. In vitro culture conditions such as culture medium, photoperiod or phytohormone ratio can be adjusted to promote somatic embryogenesis or organogenesis from induced callus [15]. The molecular pathway of shoot regeneration primarily includes phytohormones and genes related to shoot apical meristem [16]. The expression of these genes appears to be regulated at transcriptional and post-transcriptional levels. Many relevant transcripts are overseen by microRNAs (miRNAs) that have been shown to act as signals limiting gene expression in specific plant tissues [17,18,19,20]. Another example would be the study concerned with regulatory miRNA analysis in *Taxus* cells. These miRNAs regulate a series of genes involved in various pathways, including transcription factors, methyltransferases and functional enzyme genes. Genes targeted by miRNAs were correlated with pathways such as oxidative phosphorylation, RNA polymerase, purine and pyrimidine metabolism, plant hormone and signal transduction. These results indicate that miRNAs are one of the essential regulators of primary metabolism during long-term subculture [21]. Further results suggest that increasing the biosynthesis of taxol and other secondary metabolites is an active regulatory measure of calli to adapt to the heterotrophic culture, and this alteration mainly involved direct and indirect miRNA-induced transcriptional reprogramming. These results expand the understanding of the relationships among the metabolism of biological substances, the biosynthesis of secondary metabolites and defense systems [22].

Moreover, miRNA genes, as well as their biogenesis, are susceptible to aberrant regulation in vitro [23]. An example of such regulation was described for micropropagated strawberries; during in vitro culture, the miR156 was up-regulated, whereas other miRNAs (miR164, miR172 and miR390) were down-regulated [24]. Differentially regulated miRNAs are involved in many fundamental biological processes. Thus, under in vitro conditions, various miRNAs even from the same family may differ in their expression.

With the availability of next generation high-throughput sequencing (NGS) technology, thousands of miRNAs have been identified across the plant kingdom [25,26]. Many of them are conserved among plant species, whereas others are specific to certain species or even plant lines [27,28]. Conserved miRNAs do not necessarily show the same expression or pattern in different species or even at different stages of development within a species [27,29].

Plant miRNAs are typically 20–24 nt and act on targets through post-transcriptional gene silencing (PTGS), either by transcript cleavage or by translational repression [30]. The miRNAs are by far the best characterized class of sRNAs. They are involved in a series of biological processes, such as growth and development [31,32], hormone signal transduction [22,33] and response to abiotic and biotic stresses [34,35].

For cucumbers, miRNAs from leaf, root, stem and phloem exudates were analyzed in three independent high-throughput sequencing studies [36,37,38]. They identified 31, 29 and 25 known and 49, 2 and 7 novel families of miRNAs in cucumber plants. Recently, the regulatory roles of miRNAs and their targets, which are modules in cucumber fruit expansion, described the identification of 1253 known and 1269 new miRNAs from nine cucumber small RNA (sRNA) libraries by high-throughput sequencing [39].

In this study, we used three somaclonal lines (S1, S2 and S3) that differed phenotypically from the cultivar ‘Borszczagowski B10’ (the progenitor line) and were obtained by different regeneration methods. The S1 line showed a mosaic phenotype (the combination of small yellow and irregular large silvery spots on the leaves). The S2 line showed an altered fruits phenotype, which is light green, glossy, without a waxy coat and lacking typical wards and netting. The S3 line produced shoot apices yellow-green in color. The phenotypic differences in the somaclonal lines are predominantly associated with the constituent factors and explants from which the culture was initiated: direct leaf regeneration, leaf callus regeneration and embryogenic suspension culture (see details in the Materials and Methods section).

In an earlier work, we attempted to explore the variability between the genomes [40] and transcriptomes profile [41]. We suggested that differential gene expression may be caused by a polymorphism in the genic region and may also be a result of interaction among molecular networks [41]. We hypothesize that, between the somaclonal lines and the reference B10 line, there are miRNAs with differential expression, and our aim was to find the specific differentially regulated miRNAs and their targets and to indicate significant biological processes in somaclonal variations. We performed sequencing of sRNA profiles and pointed out differentially regulated miRNAs and their targets. After functional annotation and gene ontology classification of targets, the most abundant terms were described. Further, we checked the molecular interaction of target proteins with proteins encoded by genes with polymorphism, and proteins encoded by genes with differential expression with genes. In summary, we identified differential miRNAs and targets specific to somaclonal lines and also common to all lines. This is interesting, given that the somaclonal lines came/originated from different experiments. Additionally, we created molecular networks combining omic levels from DNA-seq through mRNA-seq, sRNA-seq and in silico protein–protein interaction (PPI). Such molecular network imaging is a new approach, especially for plants, and we are the first to show this for somaclonal variation.

## 2. Results

### 2.1. Overall Scheme of the Analysis

**Figure 1:**
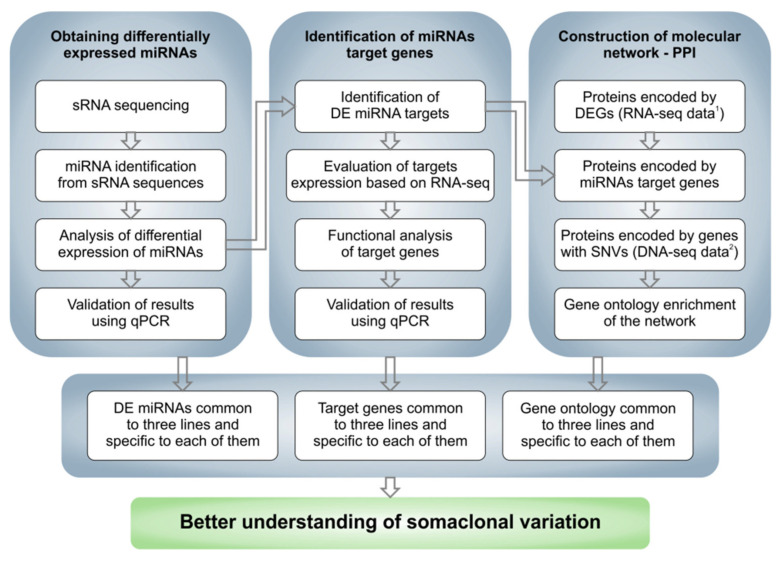
Overall scheme of the performed analysis. ^1^ RNA-seq data come from Pawełkowicz et al., 2021 [41]; ^2^ DNA-seq data come from Skarzyńska et al., 2020 [40].

The analysis performed in the experiment included several consecutive steps, starting with the analysis of the raw sequencing data. A general scheme showing the successive steps is shown in Figure 1.

### 2.2. Overview of sRNA Sequencing

**Figure 2:**
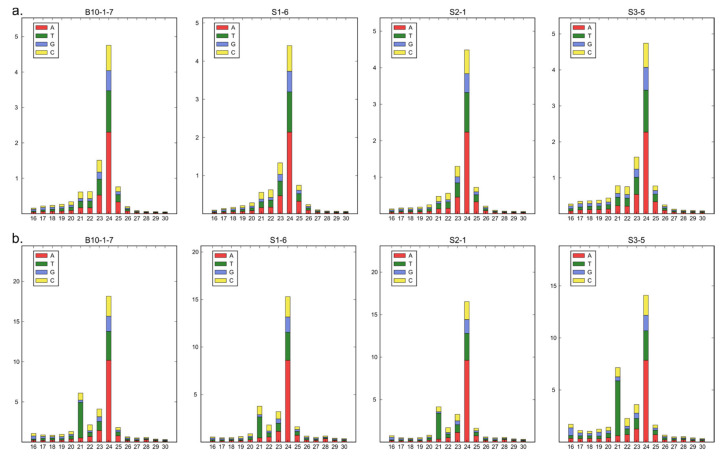
(**a**,**b**) Length distribution of sRNA sequences of three somaclonal lines (S1, S2 and S3) and control B10 line.

**Figure 3:**
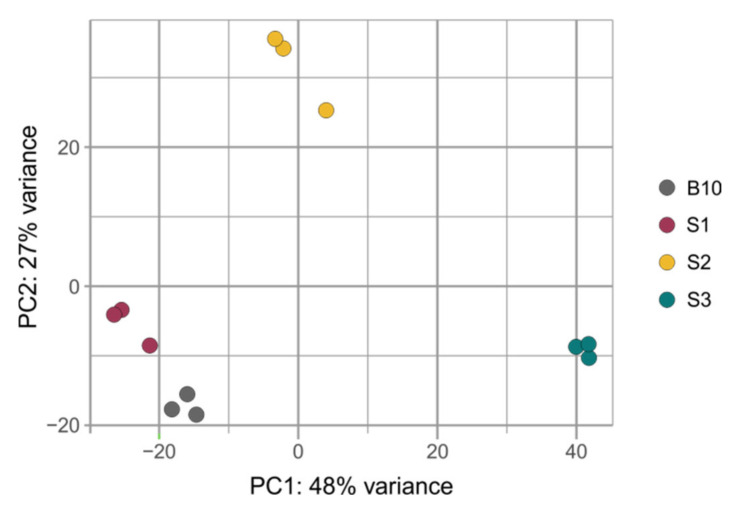
PCA plot showing distances between samples of three somaclonal lines (S1, S2 and S3) and control B10 line after sRNA-seq.

sRNAs can influence complex molecular networks and certainly play a key role in a variety of regulatory pathways involved in many plant developmental processes. We performed sRNA sequencing from small fruits (two weeks after pollination) from three cucumber somaclonal lines (S1, S2 and S3) and a control B10 line. A total of 12 sRNA libraries were constructed (three repetitions per sample). A total number of reads after high-throughput sequencing ranged between 52 and 65 million, with an average Phred quality of 37 (Table 1). The 18–25 nt reads were annotated and the length distribution was analyzed (Figure 1). More than 80% of the total redundant sRNA were between 21 and 24 nt in length, which is the most typical length of sRNA in plants. Fragments of 24 nt were most abundant in each cucumber fruit library in all sRNA profiles analyzed along with redundant (S1-47.48%; S2-48.97%; S3-39.48% and B10-45.53%) and non-redundant (S1-44.38%; S2-47.49%; S3-43.07% and B10-47.82%) pools. In all analyzed lines, the distribution of the number and length of the reads were similar (Figure 2). Principal component analysis divided sRNA samples based on their similarity into separate clusters (Figure 3).

### 2.3. miRNA Identification and Differential Expression

The first step in the analysis was to identify miRNA sequences among the sRNA sequencing results. For this purpose, ShortStack software [17] was used. This tool allowed us to create a list of potential sequences that could be classified as miRNAs to Y and N15 status (Table S1).

**Figure 4:**
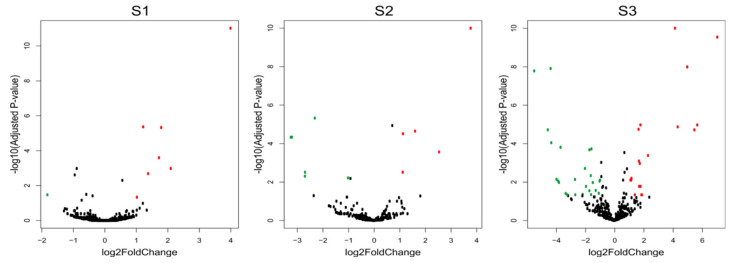
Volcano plots of differentially expressed miRNA in three cucumber somaclonal lines (S1, S2 and S3) versus control B10 line. Green dots represent significant down-regulated miRNAs, red dots represent significant up-regulated MiRNAs, and black dots represent no significant miRNA expression.

**Figure 5:**
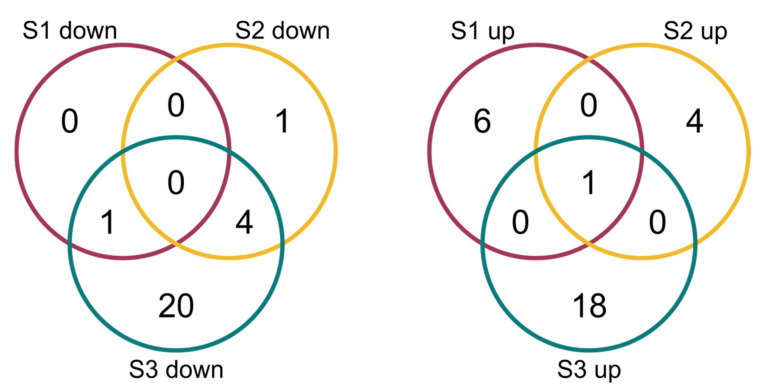
Venn diagrams of differentially expressed miRNAs in three cucumber somaclonal lines (S1, S2 and S3) in comparison to B10 line.

**Figure 6:**
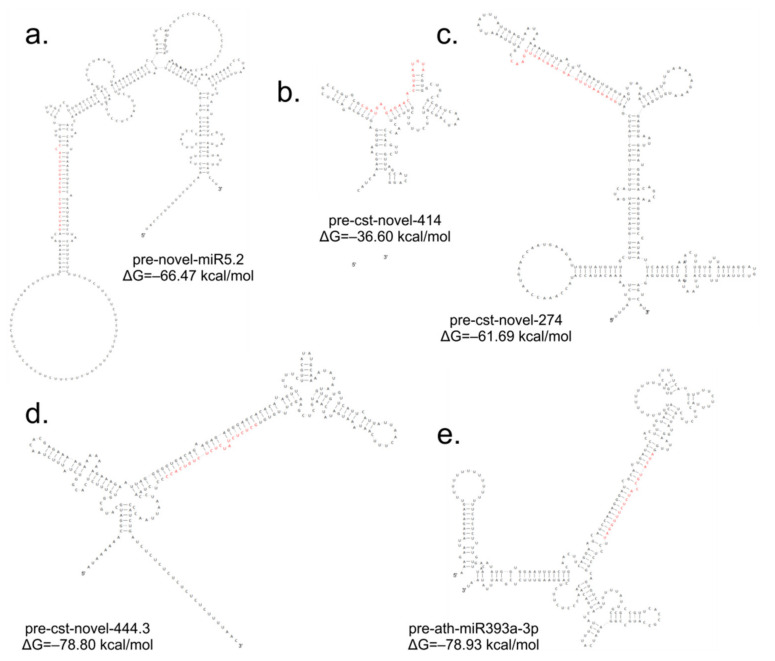
Stem-loop structures of the selected cucumber pre-miRNAs: (**a**) pre-cst-novel-miR5.2; (**b**) pre-cst-novel-miR414; (**c**) pre-cst-novel-miR274; (**d**) pre-cst-novel-miR444.3; (**e**) pre-ath-miR393a-3p. Red letters represent miRNA. Structures with the lowest free energy are presented, which reflects the most stable miRNA structures.

**Figure 7:**
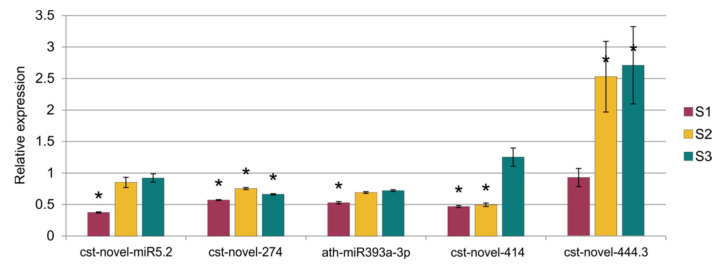
Results of the relative expression analysis of five miRNAs. Statistically important differences are marked with asterisks.

The comparison of the miRNA expression profiles of the S1, S2 and S3 lines with the control B10 line revealed differences in miRNA expression (Table 2) with parameters as an adjusted *p*-value cutoff 0.05 and a Log_2_FC (Fold Change) ≥ 1.0 (Figure 4). There were eight miRNAs in S1, one of which was down-regulated and seven of which were up-regulated. In S2, there were 10 miRNAs, five of which were down-regulated and five of which were up-regulated. The highest number of variable expressions of the miRNAs was observed in the S3 line, and it was 44 miRNAs, out of which 25 were down-regulated and 19 were up-regulated (Figure 5, Table S1). The miRNAs were aligned to the miRBase with *Cucumis* *sativus*, *Cucumis melo*, *Arabidopsis thaliana*, *Arabidopsis lyrata*, *Brassica napus*, *Brassica rapa*, *Medicago truncatula*, *Lotus japonicus* and other plant species. The annotation of differential miRNAs showed that, of all miRNAs, only 14 had database equivalents, and the rest of the miRNAs were identified as new (cst-novel-miR) (Table S1). In the S1 line, only one up-regulated miRNA (ath-miR393a-3p) was similar to those in the databases, and in the S2 line, one was down-regulated (cme-miR169l), and four were up-regulated (cme-miR167c, cme-miR394a, cme-miR171e, ath-miR393a-3p). In the S3 line, the two known miRNAs were down-regulated (cme-miR169l, cme-miR156b), and six were up-regulated (cme-miR164b, cme-miR166b, cme-miR396e, cme-miR390d, cme-miR169f, ath-miR393a-3p). Primary transcripts (pri-novel-miRNAs) form complicated, stable secondary structures, from which pre-miRNA and finally mature miRNAs are cleaved (Figure 6 and Supplementary Figure S1). Experimental confirmation of the presence of the five miRNAs was carried out by a two-step RT-qPCR method: the first step was two-tailed RT with target-specific primers, and the second step was a standard qPCR reaction (Figure 7, Table S2). The cst-novel-miR5.2 and ath-miR393a-3p were significantly down-regulated in the S1 line, but no changes were observed in the S2 and S3 lines. A significant decrease in cst-novel-miR274 expression was observed in all somaclonal lines. The cst-novel-miR414 presented a lower expression in the S1 and S2 lines and a higher expression in the S3 line, but it was not statistically significant. The cst-novel-miR444.3 expression level was significantly higher in the S2 and S3 lines.

### 2.4. Identification of Target Genes

**Figure 8:**
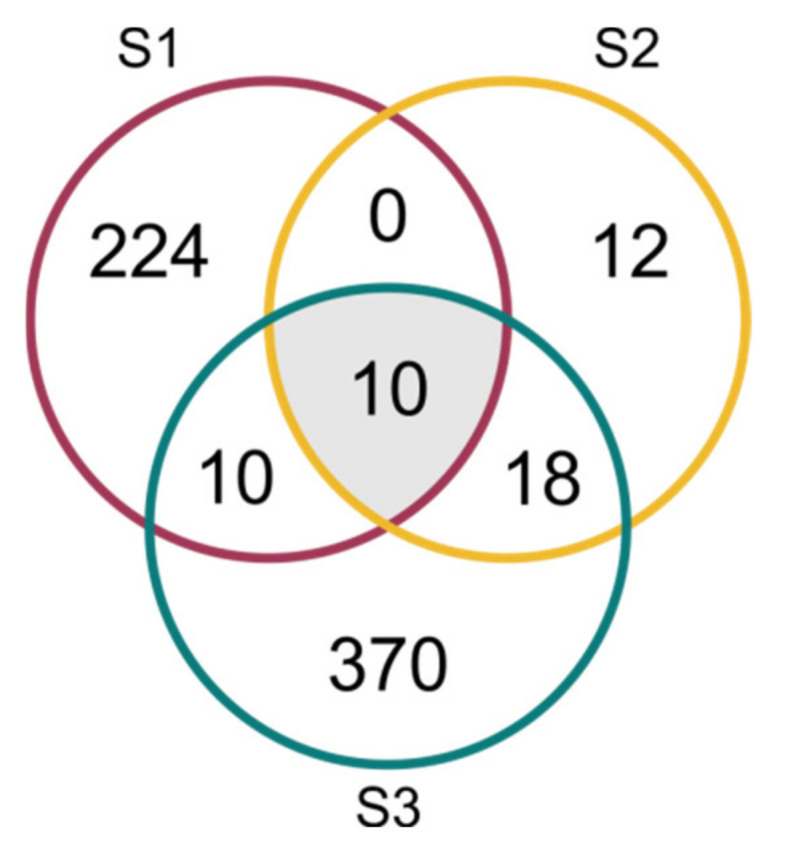
Venn diagram of targeted genes (with maximum expectation value ≤ 3) in three cucumber somaclonal lines (S1, S2 and S3).

A total of 2886 mRNAs (including isoforms) encoded by 644 genes were identified as predicted in-silico miRNA targets, including 689 targets coded by 113 genes targeted by known miRNAs, and 2284 targets coded by 564 genes targeted by novel miRNAs. In total, there were 560 mRNAs (244 genes), 283 mRNAs (40 genes) and 2400 mRNAs (408 genes) for the S1, S2 and S3 lines, respectively. The identified genes were targets to one known and six novel miRNAs in the S1 line, five known and three novel miRNAs in the S2 line, and eight known and 35 novel miRNAs in the S3 line. The annotation of the targets is listed in Table S1. Ten genes detected as targets for miRNAs were common to all three lines, and all of them were targets to ath-miR393a-3p (Figure 8, Table 3). The degradome PAREsnip analysis confirmed 173 targets in S1, 95 targets in S2 and 227 targets in S3. The same analysis confirmed one miRNA in S1, four miRNAs in S2 and 13 miRNAs in S3 (Table S3).

**Figure 9:**
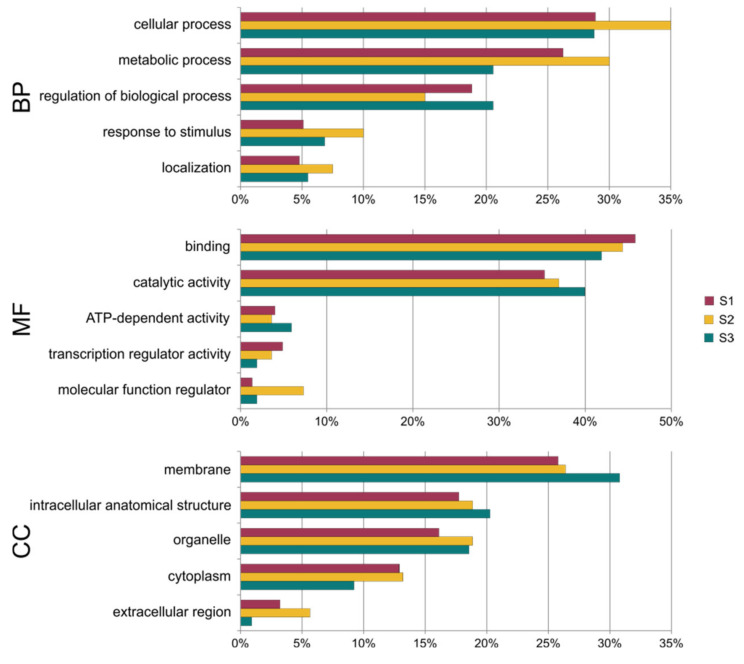
Percentage of GO terms assigned to target genes in three cucumber somaclonal lines (S1, S2 and S3). BP–biological process, MF–molecular function, CC–cellular compartment.

All detected differentially regulated miRNAs had targets. The miRNA target genes were classified by gene ontology into three categories: biological process (BP), metabolic function (MF) and cellular component (CC) (Figure 9, Table S4). There were 44 genes, 38 genes and 381 genes in the S1 line, the S2 line and the S3 line, respectively. Of the genes, 79–80% were annotated with at least one GO term. Figure 9 shows the five most abundant terms in the biological process, molecular function and cellular component categories. A detailed analysis of the GO assignment is presented in Table S4. In the biological process category, the most abundant terms were common to all of the somaclonal lines, and those were: cellular process (29–35% of genes), metabolic process (21–30%) and regulation of the biological process (15–29%). Many genes were also classified to response to stimulus (5–10%) and localization (5–8%) terms. In the S1 and the S3 line, many genes were assigned with multicellular and developmental process terms. In the cellular component category, there were also similar terms in all three lines. The membrane and its internal component (26–31% of genes), intracellular structure (16–20% of genes), organelles (16–18%) and cytoplasm (9–13%) were the most abundant terms. The most common terms in the molecular function category were binding (42–46% of genes) and catalytic activity (35–40%). Other highly prevalent terms were ATP-dependent activity (4–6%) and transcription regulator activity (2–5%). In the S3 line, a large set of genes was classified with the molecular function regulator term (7%).

**Figure 10:**
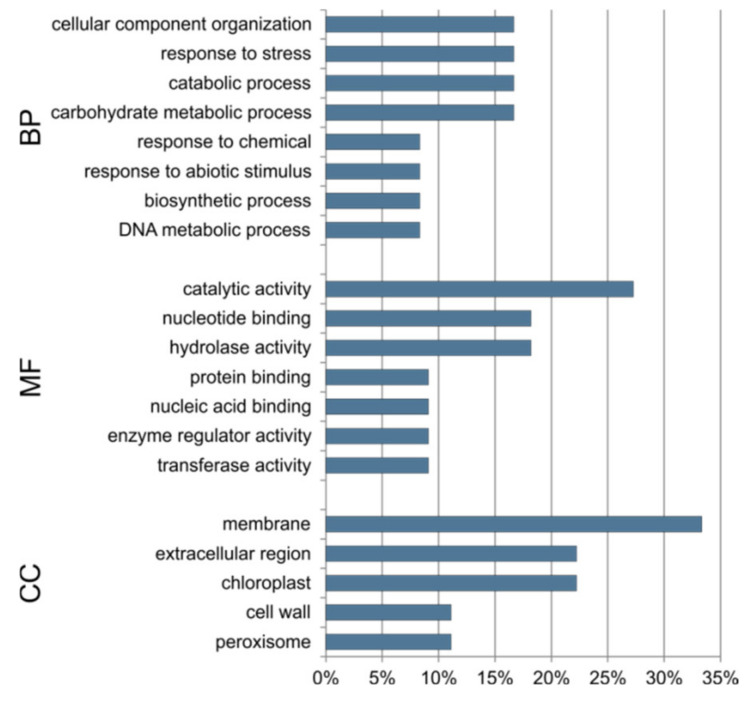
Percentage of GO terms assigned to 10 target genes that are common to all three cucumber somaclonal lines (S1, S2 and S3). BP—biological process, MF—molecular function, CC—cellular compartment.

The 10 target genes common to the three lines are responsible for cellular component organization, response to stress, and catabolic and carbohydrate metabolic processes. They show catalytic and hydrolase activity and binding of the nucleic acid and proteins. According to enrichment, the common target genes are related to membrane, cell wall, chloroplast and peroxisome (Figure 10).

**Figure 11:**
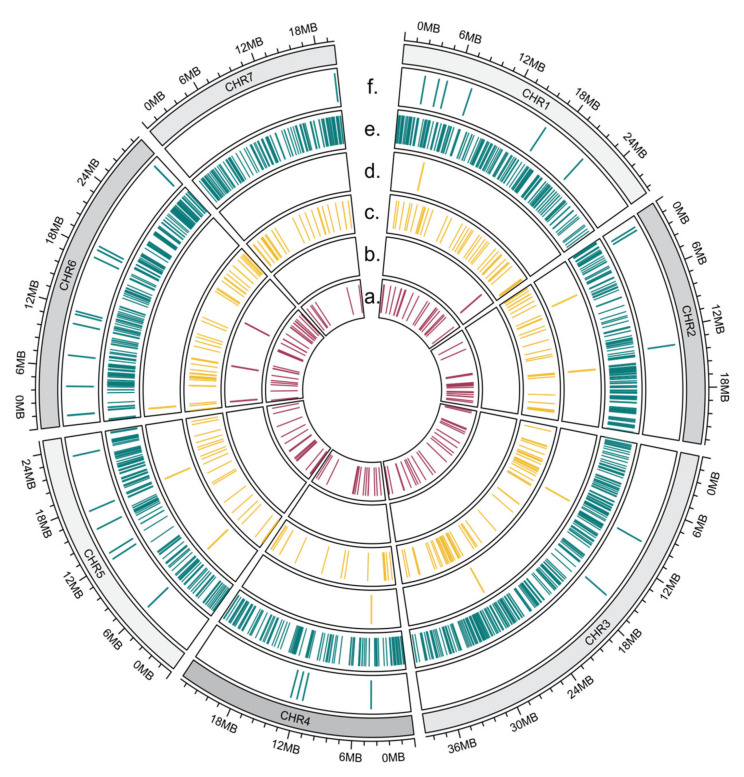
Circular map of chromosome distribution of differentially expressed miRNA genes (**b**,**d**,**f**) and their targets (**a**,**c**,**e**). Line S1 (**a**,**b**), line S2 (**c**,**d**), line S3 (**e**,**f**).

We created a genomic map and marked the chromosome localization of differentially expressed miRNA genes and their targets for each somaclonal line separately. Not all of the miRNAs are shown on the map, as not all of the reference genome contigs are assigned to the chromosomes. On the presented map, 50% of the miRNAs from the S1 line (Figure 11b), 92% of the miRNAs from the S2 line (Figure 11d) and 81% of the miRNAs from the S3 line (Figure 11f) are shown. An analysis of the target gene distribution on cucumber chromosomes (Figure 11a,c,d) showed that they are distributed evenly on seven chromosomes. The position of target genes and the position of the miRNA genes that regulate their expression seem to not be related.

### 2.5. Target Expression Profiles Based on Transcriptome Data

To profile the expression of targets, transcriptome sequencing was performed using the same samples that were used for miRNA sequencing. The data expression level of targets was extracted from the transcriptome data [41].

Transcripts were considered differentially expressed in somaclonal lines relative to the control line B10 when the log_2_Fold Change was >0.5 and the adjusted *p*-value was >0.05. A total of 40 transcripts, which are targets for miRNAs, are differentially expressed in the three somaclonal lines. Among them, four targets of two novel miRNAs and one known miRNA were in the S1 line. There were three up-regulated targets and one down-regulated target (Table 4). In the S2 line, there was one up-regulated transcript targeted by one known miRNA (Table 4). In the S3 line, there were 35 differentially expressed transcripts targeted by 12 miRNAs, among which seven were novel and five were known miRNAs (Table 4). Sixteen targets were down-regulated, and 10 targets were up-regulated.

**Figure 12:**
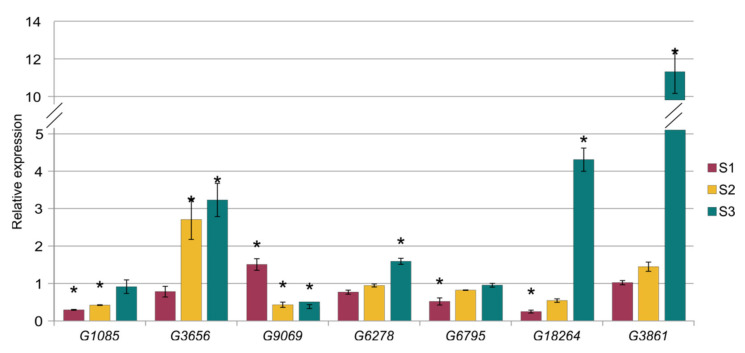
Results of the relative expression analysis of cucumber genes that are targets to the miRNA. Statistically important differences are marked with asterisks.

We also performed relative expression analysis for seven genes that are targeted by ath-miR393a-3p (*G1085* and *G3656*) and cst-novel-miR5.2 (*G8545* and *G9069*), cst-novel-miR242 (*G6795*), cst-novel-miR9 (*G18264*), cst-novel-miR19 (*G3861*) and cme-MIR394a (*G6278*). The expression of the *G1085* gene was significantly lower in the S1 and the S2 lines, whereas *G3656* gene expression was 2.7 and 3.2 times higher in lines S2 and S3, respectively, than in the control line. However, the other target gene of cst-novel-miR5.2, *G9069,* was up-regulated in the S1 line and down-regulated in the S2 and S3 lines, and all changes are significantly important (Figure 12, Table S2). The expression of the *G6795* gene was significantly lower in the S1 line. The expression of the *G18264* gene was also significantly lower in the S1 line, but 4.3 times higher than the control in the S3 line. *G3861* gene expression was 11.3 times higher in the S3 line than in the control B10 line. The relative expression level of the *G6278* gene was also significantly higher in the S3 line (Figure 12, Table S2). The expression results obtained by qPCR were in accordance with results obtained previously by RNA-seq analysis (Table S2).

### 2.6. Modeling of Interaction Regulatory Networks

We constructed a molecular network consisting of three types of protein data. The first group included in the network of protein–protein interaction (PPI) were proteins coded by target genes for miRNA, the second type were proteins coded by differentially regulated genes described in detail in Pawełkowicz et al. [41], and the third set were proteins coded by genes (pointed by genome comparisons of the somaclonal lines with the B10 genome) in which polymorphisms of high importance in the context of the structure of the emerging protein have occurred [40]. The protein interaction networks were constructed with Cytoscape with the STRING application. As input, we used a total of 581, 491 and 640 identifiers for the S1, S2 and S3 lines, respectively. These included 44 miRNA targets, 418 differentially expressed genes (DEGs) and 119 genes affected by single nucleotide variants (G_SNVs) for the the S1 line; 36 targets, 365 DEGs and 90 G_SNVs for the S2 line; and 331 targets, 198 DEGs and 111 G_SNVs for the S3 line (Table S5).

**Figure 13:**
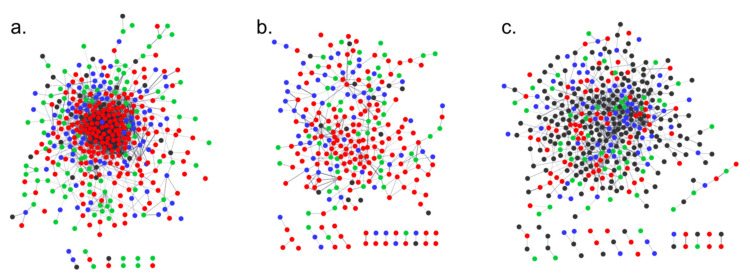
The results of STRING software analysis for protein–protein interaction network in S1 line (**a**), S2 line (**b**) and S3 line (**c**). The proteins coded by miRNA target genes are black, proteins coded by differentially regulated genes are red (up-regulated) and green (down-regulated), and proteins coded by genes with high impact polymorphism are blue.

As a result, for the S1 line, all 535 identifiers were matched in the STRING database, and 398 proteins were included in the main network, among which there were proteins encoded by 27 target genes, 288 DEGs and 83 G_SNVs. Fourteen proteins were involved in six smaller tri- and di-component networks (Figure 13, Table S5). The rest of the proteins were singletons. In the S2 line, 426 proteins were found. The main network consisted of 249 proteins, among which there were 14 targets, 181 DEGs and 54 G_SNVs. There were also 12 smaller networks containing 30 proteins. The remaining proteins were singletons (Figure 13, Table S5). In the S3 analysis, 604 proteins were matched in the STRING databases. The main network consisted of 387 proteins, among which there were 204 targets, 118 DEGs and 65 G_SNVs. The 42 proteins were grouped into 13 smaller networks. The rest of the proteins were assigned as singletons (Figure 13, Table S5).

The trend of the node degree distribution (NDD) in each network shows that most nodes have a relatively small degree, but a few nodes will have very large degrees, being connected to many other nodes (Figure S2a). The neighborhood connectivity (NC) spans over a range of values wider than the standard nodal degree, resulting in a statistically more reliable classification of infrastructure networks (Figure S2b). Plots for both NDD and NC values in the three analyzed lines indicate that the infrastructure of PPI networks are real.

We performed networks enrichment with regard to gene ontology and UniProt Keywords (Table S6). Enrichment of the molecular network in the S1 line showed that most of the terms that were linked in the cellular components group were related to plastids and their compartments. Proteins assigned to molecular function were connected with transmembrane transporter activity, oxidoreductase activity and ion binding. In the biological processes category, the most enriched terms were connected to biosynthetic processes of heme, chlorophyll and porphyrin-containing compounds, and they were also connected to a response to the absence of light. The most numerous Uniprot Keywords were porphyrin, chlorophyll and heme biosynthesis.

The molecular network enrichments in the S2 line revealed that most of the terms that were connected in the cellular component group were connected with cells and membranes. Proteins assigned to molecular function were connected with DNA binding, transcription factor activity, catalytic activity and molecular function regulator. In the category of biological processes, the most enriched terms were connected with different processes related to development of shoot systems and anatomical structure morphogenesis. The most numerous Uniprot keywords were as follows: amino-acid binding, calmodulin-binding, membrane and transcription regulation.

Enrichment of the molecular network in the S3 line showed that most of the terms that were connected in the cellular components group were related to chloroplast, cytosol and cytoplasm. Proteins assigned to molecular function were connected with compound binding and catalytic activity. In the category of biological processes, the most enriched terms were connected with microsporogenesis, regulation of reproduction process and shoot morphogenesis. The most common UniProt keyword was oxidoreductase.

## 3. Discussion

Somaclonal variability is a phenomenon that occurs during plant-regeneration in in vitro cultures. It is usually undesirable because it affects plant variability, but sometimes the new traits obtained are desirable and can provide a new source for plant breeding. The phenomenon of somaclonal variation is very complex and still poorly understood. In an earlier work, we attempted to explore the variability between the genomes of somaclonal lines (S1, S2 and S3) compared to the reference B10 line [40]. The total number of obtained polymorphisms differed from 7591 to 44510. Detected polymorphisms were most frequent in non-coding regions and were mainly SNPs. High-impact changes accounted for 1–3% of all polymorphisms [40]. Comparison of the transcriptome profiles of small fruits revealed 418, 36 and 273 genes that were differentially regulated in the S1, the S2 and the S3 line, respectively [41]. We suggested that the differential gene expression may be caused by polymorphism in the genic region and may also be a result of interaction among molecular networks, which triggers specific pathways. We wondered whether miRNAs play a role in somaclonal variation, whether there is any difference in miRNA profiles in the somaclonal lines compared to the B10 reference line, and whether these changes are transmitted generationally. For this purpose, we performed sequencing of sRNA profiles and identified differentially expressed miRNAs for which we found target molecules. Then, we checked bioinformatic analyses for possible interactions between proteins encoded by targets, G_SNV and DEGs. Checking such interactions seems to be important in the context of molecular network analysis and in the link to the phenomenon of somaclonal variation. microRNA molecules can be negative regulators of gene expression, reducing the stability of target RNAs or limiting their translation. However, in fact, the direction of these changes is unknown. According to the competitive endogenous RNA (ceRNA) hypothesis [42], the relationship between mRNAs and microRNAs could be reciprocal. In addition to the conventional microRNA → RNA function, a reversed RNA → microRNA logic exists, in which coding and noncoding RNA targets can crosstalk through their ability to compete for microRNA binding. RNA molecules could communicate with each other through microRNA and microRNA response sequences (MREs–miRNA response elements). The greater the number of shared MREs, the greater the level of “communication” and thus coregulation. Most miRNAs have multiple targets, and many mRNAs are targets for multiple miRNAs. The reason for a negative correlation in the expression level is frequently not clear. Additionally, many miRNAs target long non-coding RNAs, which also influence the RNA concentration. Therefore, it is difficult to predict the direction of changes in target expression and its impact on molecular networks.

Undoubtedly, miRNAs form a complex network and have key roles in many diverse regulatory pathways involved in plant development, plant health, environmental and disease responses [30,43,44]. Therefore, could miRNA be responsible for the development of somaclonal variation? Is there one pattern triggering miRNA complexes that leads to changes in regenerants? We demonstrated this through an in silico display of a PPI network in which one of the components was targets regulated by cleavage or translation inhibition by miRNAs. This has an impact on the proteome content, as seen in the molecular networks that we have performed.

The complex network theory (CNT) is becoming one of the most powerful and versatile tools to investigate, describe and understand biological systems [45]. In the last decades, CNT has had an unrestrained development, and researchers have proposed novel approaches, metrics and theories to explore and disentangle network features [46,47,48]. Protein–protein interaction analysis with the STRING tool allows for proper configuration of the network based on a database containing all available protein association evidence and prediction algorithms. Herein, we performed complex analysis of multi-omics research to elucidate the role of miRNA with target genes and its influence on protein occurrence or absence. The regulatory role of miRNA in each somaclonal line on the protein–protein networks is proven. miRNA interacts with the targets mainly in two ways: by cleavage or translation inhibition, thus causing disturbances in the abundance or structure of the resulting proteins encoded by target genes for small miRNA molecules. In our in silico research, we created networks including proteins encoded by target genes, and as shown in Figure 13, they interact with clusters encoded by genes with high impact polymorphisms [40,41] and the genes that are differentially expressed [41]. Therefore, we can conclude that miRNA plays a role in complex processes in cucumber fruit derived from the somaclonal lines. From the analyses performed, a functional picture emerges, in which the components are involved mostly in processes connected with metabolic processes, as well as the regulation of biological processes with regard to molecular function in binding and catalytic activity. We observed similar enrichment in gene ontology across all three somaclonal lines. This may indicate co-processes that occur when new pathways are created or when alternative pathways in plants lead to somaclonal changes. Further, in the cellular component category, the enrichment network analysis showed terms common to the three somaclonal lines, such as membranes, cytoplasm and organelle (especially chloroplast in the S1 and S3 lines), which indicates that the proteins of the interactome under study act mainly in these areas. Most cellular activities take place within the cytoplasm, e.g., many metabolic pathways. Movement of ions in and out of the cytoplasm is a signaling activity for metabolic processes [49]. Membranes act as major checkpoints for signal sensing and transport control. An important role in communication is played by membrane-associated proteins, which interact with intracellular processes through protein interaction networks [50]. Deciphering these signaling networks certainly provides important information for elucidating in vivo cellular regulation in somaclones, particularly membrane–protein interactions, as well as how these proteins may be related to downstream changes in gene expression, metabolism and plant physiology. It seems that the molecular networks we present illustrate how plants responded to in vitro cultures. Membranes, and more specifically receptor proteins, transmit a signal inside the cell, and there are other proteins whose role is to participate in metabolic pathways that make the cell adapt to the surrounding environment of in vitro cultures.

Returning to the question of miRNA’s role in this complex process, it seems certain that by acting on target molecules through digestion or translation inhibition, miRNAs influence signaling networks in response to the conditions in in vitro cultures. Target genes are involved in processes such as cellular and metabolic processes and their regulation, and more particularly in response to stress, multicellular organism development, biosynthetic process and transport. The most abundant terms in the category of molecular function were: catalytic activity, protein binding, binding, hydrolase and transferase activity, nucleic acid binding and transcription factor activity. This is in line with the trend described above for the terms that were the most numerous groups in the network enrichment analysis. The same applies to cellular compartments.

This connection with ontological groups also shows that proteins are involved in numerous processes. In the cells of the cucumber somaclonal lines, numerous metabolic processes take place, taking into account various biochemical changes of proteins, which may be a response to the conditions in in vitro culture. Surely, these numerous reactions are intended to adapt to the environment of the in vitro cultures and to develop reaction pathways in such a way that the plants survive.

In the molecular function category are also proteins connected with nucleic acid binding and transcription factors, which are essential for the regulation of gene expression and usually belong to members of multigene families [51]. Generally, TFs exist as modular proteins containing a DNA-binding domain that interacts with *cis*-elements of their target genes [52]. Moreover, it also consists of a protein–protein interaction domain that assists oligomerization between TFs or with other regulators [53]. We hypothesized that miRNAs could act with targets that are TF, and thus influence other genes, resulting in the changing of transcriptome profiles in somaclonal lines. The regulation of genes at transcriptional and post-transcriptional levels, which includes miRNAs and TFs as key regulatory entities, is an interesting aspect. Understanding the regulation of miRNAs and their interactions with TF can help scientists deepen their understanding of plants and the mechanism of survival in adverse environmental conditions [54] or, in this case, in an in vitro culture environment.

Noteworthy is the fact that the common miRNA ath-miR393a-3p has changed in all three somaclonal lines. This sequence belongs to the miR393 family, which is predicted to target mRNAs coding for F-box proteins and bHLH transcription factors [55]. F-box proteins are substrate-recognition components of the *S*kp1-Rbx1-*C*ul1-F-box protein (SCF) ubiquitin ligases. In plants, F-box genes form one of the largest multigene superfamilies and control many important biological functions. However, it is unclear how and why plants have acquired a large number of F-box genes [56]. The bHLH transcription factors comprise a large family in higher plants, and numerous studies have shown that bHLH-type transcription factors are involved in diverse biological processes in plant growth, development and stress responses [57]. There are a multitude of processes in which these TFs are involved: F-box and bHLH confirm that miRNAs, by reacting with these targets as TFs, can have a large impact on other genes.

Other miRNAs pointed out in our study as differentially expressed with counterparts in the miRBase data were similar to those from *Cucumis melo* [58]. Among the down-regulated miRNAs were cme-miR169l in the S2 and S3 lines and cme-miR156b in the S3 line. Among up-regulated miRNAs were: cme-miR167c, cme-miR394a and cme-miR171e in the S2 line; and cme-miR164b, cme-miR166b, cme-miR396e, cme-miR390d and cme-miR169f in the S3 line.

Many of the down-regulated miRNAs were also associated with transcription factors. The cme-MIR394a belongs to the miR394 family, which is predicted to target mRNAs coding for F-box proteins [55]. The miR156 is expressed at high levels in organs produced early in shoot development, where they repress the expression of their targets, SQUAMOSA PROMOTER BINDING PROTEIN (SBP) transcription factors [59]. In addition, miR156 was recently identified as an enhancer of the callus embryogenic potential in *Citrus sinensis* [60]. miR166b is thought to target mRNAs coding for HD-Zip transcription factors, including Phabulosa (PHB) and Phavoluta (PHV), that regulate axillary meristem initiation and leaf development [61]. The cme-MIR171e is also associated with transcription factors [55].

Several miRNAs appear to be temporally regulated during development, such as the miR167, miR169 and miR390 families during ovarian development [62], or miR164b, which targets mRNAs encoding NAC domain-containing proteins such as the cup-shaped cotyledon 2 (CUC2) required for shoot apical meristem formation [61]. Furthermore, transcription factors CUC1 and CUC2 regulated by miR164 participate in the establishment and maintenance of axillary meristem and organ boundary during embryogenesis [63,64]. Further, miR396 regulates growth-regulating factor genes [65]. Some miRNAs, including miR156 and miR169, are associated with the coordination of the relationship between development and stress responses [66].

miRNAs have been reported to be involved during in vitro culture of plants [23,67]. Differential microRNA expression has been recorded in micropropagated strawberry plantlets regenerated by tissue culture, and these have been correlated with existing differences with the phenotype [24]. During in vitro culture, the miR156 was upregulated, whereas other miRNAs (miR164, miR172 and miR390) were downregulated. Authors have suggested that the regulation of miRNAs may differ in their expression, and the regulation of miRNAs is controlled by different factors in the culture media, such as hormones allowing differentiated tissue to reach growth potential [24].

In our study with cucumber, the set of miRNAs belonging to the same families were differentially regulated similarly to those in the strawberry. However, to date, no direct relationship has been found between somaclonal variation and miRNA [68]. Further studies are required to characterize the miRNA expressed during somaclonal variation from different plants so that diagnostic miRNA associated with this phenomenon can be compiled and its possible use as a biomarker facilitated [66]. Typically, this work concerned changes in miRNAs during various periods of in vitro time cultures, with the addition of various substances to the medium, but not directly connected with somaclonal variation.

In our work, we compared miRNA profiles and connected these results with other omics studies performed on the same plant [40,41]. In a sequence similarity analysis, we identified novel and known miRNAs, that were also associated with somaclonal variation according to the literature [24]. In order to study the effects of miRNAs, we identified targets and annotated their functions and gene ontology. Further, we performed multi-omics interaction networks, using predicted in silico targets from this study and additional polymorphism data from the study of comparative genomics [40] and data from transcriptome profiling [41]. We have shown that miRNAs can have an influence on the molecular networks in the cell, by regulating various metabolic processes and influencing transcription factors that are responsible for direct gene expression. Further in-depth studies of miRNAs may shed light on essential hotspots of the regulatory pathways leading to somaclonal variation in plants.

## 4. Materials and Methods

### 4.1. Plant Material

Three cucumber somaclonal lines and one control B10 line were used in this study. The somaclones have the same genetic background, as the B10 line was a donor of explants for in vitro cultures, from which the analyzed lines were created in the later stages of development. The S1 line was obtained by direct leaf regeneration [69,70] and showed a mosaic phenotype, with irregular small yellow and silvery spots [69,70,71]. The S2 line was obtained from leaf callus regeneration. It possesses an altered fruit phenotype, which is light green, glossy, without a waxy coat, and lacking typical wards and netting [72]. The S3 line was obtained from cytokinin-dependent embryogenic suspension culture, and it possesses shoot apices that are yellow-green in color [73]. After the selection of each type of somaclone, the somaclones were self-pollinated for at least 10 generations with the maintenance of a specific phenotype that arose during in vitro cultures. The field experiment was carried out using the random blocks method. Ten plants per each line were seeded and phenotypically assessed. After self-pollination, young fruits (7 days old) were harvested, frozen in liquid nitrogen and stored at −80 °C.

### 4.2. Isolation of RNA, Library Construction and High-Throughput Sequencing of the Small RNA Fraction

Total RNA was extracted from 100 mg of tissue using the RNeasy Mini Kit (Qiagen, Valencia, CA, USA), with an additional step of DNase I treatment, in accordance with the manufacturer’s protocol. The nucleic acid concentration and quality were assessed with a NanoDrop 2000 Spectrophotometer (Thermo Fisher Scientific, Waltham, MA, USA) and via standard electrophoresis on a 1.0% (*w*/*v*) ethidium bromide-stained agarose gel to allow for RNA visualization. Of the RNA, 10 µg from each of the three biological replicates (per sample) were used for the preparation of the sRNA library TrueSeq Small RNA library Kit (Illumina, Inc. San Diego, CA, USA). Parallel sequencing was performed on an Illumina HiSeq 2000 SR50 platform (McGill University Genome Quebec Innovation Centre, Montreal, QC, Canada). We obtained 50-bp single-end sequenced reads for sRNA-seq. FastQC [74] was used to assess the quality of the short reads. The sequences generated in this study have been deposited in the Sequence Read Archive (SRA) at the National Center for Biotechnology Information under accession numbers BioProjects: PRJNA723857 and PRJNA610495.

### 4.3. Bioinformatic Assessment of the miRNA and Targets: Degradome Verification

The identification of microRNAs and other sRNAs from small RNA-Seq data was performed with ShortStack ver. 3.3 [17]. The sequence was considered to be miRNA when it was tagged Y or N15 based on Axtell’s criteria [17,18]. The clean reads were used in the BLAST [75] search against known mature miRNAs and pre-miRNAs of the miRbase (version 22.1) [76]. Differential expression analysis was performed with DESeq2 [77] using default parameters. Differentially expressed miRNAs were detected using DESeq with an adjusted *p*-value cutoff 0.05 and a Log_2_FC (Fold Change) ≥ 1.0. The in–silico predicted targets of the mature miRNA sequences were identified using psRNAtarget 2017 with the scale range 0-5 [78]. For further analysis, we used only those targets that ranged on a scale of 0–3. Functional annotation and Gene Ontology (GO) classification of the miRNA targets were carried out using Blast2GO software [79]. The obtained sequences were folded using Folder version 1.12 (RNAfold algorithm) [80]. The longest sequences that could form the stem–loop structures were used for pre-microRNA construction. The pre-microRNA structures with the lowest ΔG energy value were chosen, and the corresponding miRNA was marked in red. miRNA target interactions were confirmed using our miRNA FASTA and transcriptome files with publicly available cucumber degradome data (SRR7620953 from NCBI SRA archive) [81,82]. The small RNA Workbench ver. 3.2 (http://srna-workbench.cmp.uea.ac.uk/; 28 February 2022) [83] software package was used for this analysis with low default stringency parameters (*p*-value cut off 0.05 and 4.5 number of mismatches allowed, in category scale 0–4).

### 4.4. Integration of Multi Omics Data

Identified miRNAs and their targets were compared with previously obtained data related to creating a multi-omics molecular network. For this purpose, we used miRNA targets obtained in this study and previously obtained genomic [40] and transcriptomic data [41]. The genomic data (BioProject PRJNA563814) come from genome sequencing of the somaclonal lines and consist of selected genes affected by structural polymorphisms SNVs (Single Nucleotide Variants) [40]. The transcriptome data (BioProjects 578634 and 578623) contain the differentially expressed genes (DEGs) from cucumber fruits [40]. As a reference cucumber, genome B10v3 was used (GenBank LKUO00000000) [84]. The STRING algorithm (version 10.5) [85], using *Arabidopsis thaliana* as a model, was applied for analysis of the possible interactions between protein–protein interaction (PPI) encoded by DEGs, G_SNVs and miRNA targets (Table S4). We used Cytoscape string APP (version 3.7.2) [86] to edit the layout of our map. Each node was color-coded based on the data type. The networks enrichment was performed with regard to gene ontology and UniProt Keywords (Table S5).

### 4.5. qPCR Analysis of Expression of miRNA and Their Target Genes

Total RNA was isolated with the miRNeasy Micro Kit (Qiagen), which is effective for total RNA and miRNA, according to standard protocol. Reverse transcription was performed with the High-Capacity cDNA Reverse Transcription Kit (Thermo Fisher Scientific, Waltham, MA, USA) according to the manufacturer’s protocol. For cDNA synthesis, 1 μg of the total RNA was used. cDNA synthesis of miRNA was performed using the two-tailed RT target-specific primers. All primers for miRNA qPCR were designed according to the two-tailed RT-qPCR method [87]. miRNAs for the analysis were chosen randomly from miRNAs differentially expressed in all lines. For target validation, target genes of the chosen miRNAs that were differentially expressed at significant levels were chosen. Quantitative PCR analysis of miRNA and target transcripts was performed with three biological and three technical replicates using 3 µL of diluted (1:5) cDNA, Power SYBR Green Master Mix (Thermo Fisher Scientific, Waltham, MA, USA) and the Applied Biosystems 7500 Real-Time PCR System (Thermo Fisher Scientific, Waltham, MA, USA). A melting curve analysis was completed immediately after the qPCR. Relative expression levels were determined according to the 2^−ΔΔCt^ method, and statistical significance analysis was performed using REST2009 software. For the relative expression analysis of miRNA, the U6 snRNA was used as a reference, and for the analysis of target genes, the *CACS* gene was used as a reference. The full list of primers used in the RT-qPCR analysis is shown in Table S4.

## 5. Conclusions

In the analysis, we confirmed our hypothesis of the existence of differentially expressed miRNAs between the somaclonal lines and the reference B10 line of *Cucumis sativus*. We conclude that miRNAs have a role in somaclonal variation, influencing targets by cleavage or translation inhibition. Predicted in-silico targets are mostly connected with metabolic processes, response to stress, multicellular organism development and biosynthetic process. The analysis of the targets’ functions revealed that most of them were classified to catalytic activity, binding, transferase activity and transcription regulation activity. In the cellular component category, the most abundant terms were: membrane, intracellular, cytoplasm and organelle. The connection with ontological groups shows that target proteins are involved in numerous processes. In the cells of the cucumber somaclonal lines, numerous metabolic processes take place to react and adapt to the environment of the in vitro cultures. We identified several novel and known miRNAs, and the target molecules of the latter are mostly transcription factors influencing many other genes. We also checked bioinformatic analyses for possible interactions at the level of target proteins, genes affected by polymorphism and genes that are differentially regulated. Herein, for the first time, we showed the complexity of processes by molecular imaging of interaction from different omic’s levels. We hypothesize that miRNAs could indirectly affect molecular networks and could play a role in many diverse regulatory pathways leading to somaclonal variation.

## Figures and Tables

**Table 1:** Statistics of sRNA-sequencing analysis of three somaclonal lines (S1, S2 and S3) and control B10 line.

Name	No of Reads	No of Bases	Average Quality	No of Redundant Reads	No of Non-Redundant Reads
B10-1 replica 1	54,414,580	2,720,729,000	37	49,609,519	10,631,119
B10-1 replica 2	52,662,855	2,633,142,750	38	47,499,178	10,183,587
B10-1 replica 3	53,443,079	2,672,153,950	37	49,140,803	10,609,010
S1 replica 1	59,957,197	2,997,859,850	37	56,996,268	12,906,453
S1 replica 2	65,970,225	3,298,511,250	37	61,387,885	13,627,221
S1 replica 3	55,958,724	2,797,936,200	38	50,632,900	10,480,173
S2 replica 1	54,754,145	2,737,707,250	37	50,586,963	10,037,716
S2 replica 2	55,012,115	2,750,605,750	37	51,853,481	12,727,260
S2 replica 3	63,929,895	3,196,494,750	37	60,299,086	11,541,983
S3 replica 1	54,014,273	2,700,713,650	37	49,956,937	12,497,879
S3 replica 2	57,712,663	2,885,633,150	37	52,286,044	11,925,631
S3 replica 3	54,880,025	2,744,001,250	37	51,545,852	12,237,325

**Table 2:** Differentially expressed miRNAs in three somaclonal lines (S1, S2 and S3) in comparison to B10 line.

ID	log_2_FC	Adj. *p* Value	Expression	Line
cst-novel-miR114	−1.803	3.37 × 10^–2^	down	S1
ath-miR393a-3p	1.039	4.61 × 10^–2^	up	S1
cst-novel-miR414	1.238	4.32 × 10^–6^	up	S1
cst-novel-miR403	1.395	2.07 × 10^–3^	up	S1
cst-novel-miR400	1.740	2.53 × 10^–4^	up	S1
cst-novel-miR436	1.811	4.70 × 10^–6^	up	S1
cst-novel-miR246	2.114	1.04 × 10^–3^	up	S1
cst-novel-miR229	4.011	9.62 × 10^–12^	up	S1
cst-novel-miR486	−3.227	4.66 × 10^–5^	down	S2
cme-MIR169l	−3.184	4.66 × 10^–5^	down	S2
cst-novel-miR196.2	−2.677	4.96 × 10^–3^	down	S2
cst-novel-miR356	−2.672	3.08 × 10^–3^	down	S2
cst-novel-miR320	−2.291	4.72 × 10^–6^	down	S2
cme-MIR167c	1.140	3.08 × 10^–3^	up	S2
cme-MIR394a	1.150	3.09 × 10^–5^	up	S2
ath-miR393a-3p	1.623	2.25 × 10^–5^	up	S2
cme-MIR171e	2.560	2.72 × 10^–4^	up	S2
cst-novel-miR377	3.790	1.01 × 10^–10^	up	S2
cst-novel-miR153	−5.474	1.65 × 10^–8^	down	S3
cst-novel-miR196.2	−4.543	1.92 × 10^–5^	down	S3
cme-MIR169l	−4.350	1.23 × 10^–8^	down	S3
cst-novel-miR486	−4.304	8.98 × 10^–5^	down	S3
cst-novel-miR222	−3.946	7.18 × 10^–3^	down	S3
cst-novel-miR438	−3.945	7.18 × 10^–3^	down	S3
cst-novel-miR114	−3.827	8.94 × 10^–3^	down	S3
cst-novel-miR261	−3.775	1.06 × 10^–2^	down	S3
cst-novel-miR356	−3.668	1.54 × 10^–4^	down	S3
cst-novel-miR319	−3.320	3.90 × 10^–2^	down	S3
cst-novel-miR394	−3.210	4.61 × 10^–2^	down	S3
cst-novel-miR377	−3.207	4.61 × 10^–2^	down	S3
cst-novel-miR274	−2.680	7.30 × 10^–3^	down	S3
cst-novel-miR39	−2.662	4.61 × 10^–2^	down	S3
cst-novel-miR280	−2.152	4.38 × 10^–2^	down	S3
cme-MIR156b	−1.996	1.96 × 10^–3^	down	S3
cst-novel-miR94	−1.925	1.67 × 10^–2^	down	S3
cst-novel-miR346	−1.694	2.12 × 10^–4^	down	S3
cst-novel-miR156	−1.694	2.84 × 10^–2^	down	S3
cst-novel-miR195.2	−1.597	4.61 × 10^–3^	down	S3
cst-novel-miR104	−1.575	4.52 × 10^–2^	down	S3
cst-novel-miR242	−1.550	1.87 × 10^–4^	down	S3
cst-novel-miR167	−1.463	1.06 × 10^–2^	down	S3
cst-novel-miR452	−1.257	2.73 × 10^–2^	down	S3
cst-novel-miR9	−1.051	3.82 × 10^–2^	down	S3
cst-novel-miR149	1.120	7.88 × 10^–3^	up	S3
ath-miR393a-3p	1.171	7.88 × 10^–3^	up	S3
cst-novel-miR5.2	1.186	6.35 × 10^–3^	up	S3
cst-novel-miR26	1.423	4.61 × 10^–2^	up	S3
cme-MIR166b	1.674	1.79 × 10^–5^	up	S3
cst-novel-miR138	1.700	8.01 × 10^–4^	up	S3
cme-MIR396e	1.717	1.67 × 10^–2^	up	S3
cst-novel-miR358	1.760	1.08 × 10^–3^	up	S3
cme-miR164b	1.804	1.07 × 10^–5^	up	S3
cst-novel-miR113.2	1.810	1.67 × 10^–2^	up	S3
cst-novel-miR444.3	1.866	4.61 × 10^–2^	up	S3
cst-novel-miR120	1.912	4.61 × 10^–2^	up	S3
cme-MIR390d	2.323	4.13 × 10^–4^	up	S3
cme-MIR169f	4.171	9.86 × 10^–11^	up	S3
cst-novel-miR207	4.356	1.34 × 10^–5^	up	S3
cst-novel-miR394.2	5.011	1.02 × 10^–8^	up	S3
cst-novel-miR318	5.495	1.92 × 10^–5^	up	S3
cst-novel-miR19	5.697	1.07 × 10^–5^	up	S3
cst-novel-miR441	7.069	2.86 × 10^–10^	up	S3

**Table 3:** Description of 10 target genes that were common to all three somaclonal lines and their miRNAs. ^C^—regulation of gene expression by cleavage.

Target	Biotype	Description	miRNA		
			S1	S2	S3
*G1085*	protein_coding	cytochrome P450	ath-miR393a-3p ^C^		
*G10994*	protein_coding	peroxisome biogenesis protein	ath-miR393a-3p ^C^		
*G15867*	protein_coding	peroxisome biogenesis protein	ath-miR393a-3p ^C^		
*G16626*	protein_coding	chaperone protein	ath-miR393a-3p ^C^		
*G2235*	protein_coding	OTU domain-containing protein	ath-miR393a-3p ^C^		
*G2374*	protein_coding	Putative methyltransferase	ath-miR393a-3p ^C^		
*G2520*	protein_coding	CAS1 domain-containing protein	ath-miR393a-3p ^C^		
*G3656*	protein_coding	pectinesterase	ath-miR393a-3p ^C^		
*G5603*	protein_coding	NADH dehydrogenase-like	ath-miR393a-3p ^C^		
*G6384*	protein_coding	GYF domain-containing protein	ath-miR393a-3p ^C^		

**Table 4:** Differentially expressed genes that are targets of differentially expressed miRNAs in lines S1, S2 and S3.

miRNA	Target	log_2_FC	Adj. *p* Val.	Description	Line
cst-novel-miR114	*G17365.T39 ^C^*	−1.92	4.68 × 10^–3^	EamA-like transporter family	S1
ath-miR393a-3p	*G16626.T6*	0.73	3.86 × 10^–2^	chaperone protein	S1
ath-miR393a-3p	*G2374.T7 ^C^*	1.82	1.02 × 10^–6^	Putative methyltransferase	S1
cst-novel-miR246	*G5022.T1 ^C^*	2.15	2.80 × 10^–2^	flavin-containing monooxygenase	S1
ath-miR393a-3p	*G3656.T2 ^C^*	1.66	1.65 × 10^–2^	pectinesterase	S2
cme-MIR396e	*G10545.T4 ^C^*	−0.73	1.82 × 10^–3^	CW-type Zinc Finger | F-Box protein	S3
cme-MIR396e	*G10545.T5 ^C^*	−0.75	2.61 × 10^–2^	CW-type Zinc Finger | F-Box protein	S3
cst-novel-miR113-2	*G13161.T1 ^C^*	−1.90	2.46 × 10^–2^	Squamosa promoter-binding-like protein	S3
cme-MIR166b	*G13471.T1 ^T^*	−0.90	4.11 × 10^–2^	protein brittle-1, chloroplastic	S3
cst-novel-miR358	*G15151.T1 ^C^*	1.48	3.37 × 10^–2^	unknown	S3
cme-MIR390d	*G18807.T10 ^C^*	−4.09	4.72 × 10^–6^	zinc finger C3HC4 type family protein	S3
cme-MIR390d	*G18807.T14 ^C^*	−2.11	4.86 × 10^–3^	zinc finger C3HC4 type family protein	S3
cme-MIR390d	*G18807.T24 ^C^*	−1.33	1.83 × 10^–2^	zinc finger C3HC4 type family protein	S3
cme-MIR390d	*G18807.T35 ^C^*	1.38	1.39 × 10^–2^	zinc finger C3HC4 type family protein	S3
cme-MIR390d	*G18807.T43 ^C^*	1.84	4.44 × 10^–4^	zinc finger C3HC4 type family protein	S3
cme-MIR390d	*G18807.T53 ^C^*	1.45	8.43 × 10^–4^	zinc finger C3HC4 type family protein	S3
cme-MIR390d	*G18807.T61 ^C^*	1.44	3.40 × 10^–3^	zinc finger C3HC4 type family protein	S3
cme-MIR390d	*G18807.T9 ^C^*	2.66	5.22 × 10^–3^	zinc finger C3HC4 type family protein	S3
cst-novel-miR120	*G19248.T1 ^T^*	−0.66	1.66 × 10^–2^	BRCA1 C Terminus (BRCT) domain	S3
cst-novel-miR120	*G20176.T1 ^T^*	6.68	4.36 × 10^–21^	unknown	S3
cst-novel-miR120	*G20340.T1 ^T^*	−8.16	3.74 × 10^–5^	unknown	S3
ath-miR393a-3p	*G2374.T7 ^C^*	0.98	1.14 × 10^–2^	Putative methyltransferase	S3
cst-novel-miR452	*G3070.T15 ^C^*	−4.03	1.02 × 10^–12^	Inherit from NOG: expressed protein	S3
cst-novel-miR452	*G3070.T17 ^C^*	−2.27	3.06 × 10^–2^	expressed protein	S3
cst-novel-miR452	*G3070.T2 ^C^*	−2.18	4.61 × 10^–9^	Inherit from NOG: expressed protein	S3
cst-novel-miR452	*G3070.T26 ^C^*	−3.10	4.11 × 10^–2^	expressed protein	S3
cst-novel-miR452	*G3070.T28 ^C^*	−1.90	3.99 × 10^–7^	Inherit from NOG: expressed protein	S3
ath-miR393a-3p	*G3656.T1 ^C^*	1.90	1.37 × 10^–2^	pectinesterase	S3
ath-miR393a-3p	*G3656.T2 ^C^*	2.00	9.79 × 10^–4^	pectinesterase	S3
cst-novel-miR120	*G5265.T9 ^T^*	4.97	7.83 × 10^–8^	unknown	S3
cst-novel-miR26	*G6017.T3 ^C^*	0.48	4.29 × 10^–2^	unknown	S3
cst-novel-miR26	*G6017.T4 ^C^*	0.88	4.75 × 10^–2^	unknown	S3
cst-novel-miR26	*G6017.T8 ^C^*	0.78	4.30 × 10^–2^	unknown	S3
cst-novel-miR26	*G6017.T9 ^C^*	0.66	1.84 × 10^–2^	unknown	S3
cst-novel-miR5-2	*G8545.T1 ^C^*	−1.78	2.55 × 10^–6^	NAC domain-containing protein	S3
cst-novel-miR94	*G8689.T23 ^C^*	0.79	1.72 × 10^–2^	Ca-transport ATPase 9, plasma membrane	S3
cst-novel-miR94	*G8689.T26 ^C^*	0.85	1.19 × 10^–2^	Ca-transport ATPase 9, plasma membrane	S3
cst-novel-miR94	*G8689.T35 ^C^*	0.66	1.92 × 10^–2^	Ca-transport ATPase 9, plasma membrane	S3
cst-novel-miR113-2	*G8943.T2 ^C^*	2.73	1.64 × 10^–2^	squamosa	S3
cst-novel-miR5-2	*G9069.T2 ^C^*	−3.01	6.62 × 10^–3^	Protein CUP-SHAPED COTYLEDON	S3

In silico predicted inhibition: ^C^ Target regulation by cleavage, ^T^ Target regulation by translation inhibition.

## Data Availability

All clean reads generated by Illumina sequencing in this study have been deposited in the National Center of Biotechnology Information Sequence Read Archive (SRA) database (http://www.ncbi.nlm.nih.gov/sra; 12 April 2022) under the BioProject accessions PRJNA723857 and PRJNA610495.

## Supplementary Materials

The following supporting information can be downloaded at: https://www.mdpi.com/article/10.3390/ijms23084317/s1.

## References

[1] Larkin P.J., Scowcroft W.R. (1981). Somaclonal variation—A novel source of variability from cell cultures for plant improvment. Theor. Appl. Genet..

[2] Kaeppler S.M., Phillips R.L., Olhoft P., Jain S.M., Brar D.S., Ahloowalia B.S. (1998). Molecular basis of heritable tissue culture—Induced variation in plants. Somaclonal Variation and Induced Mutation in Crop Improvement.

[3] Zhang D., Wang Z., Wang N., Gao Y., Liu Y., Wu Y., Bai Y., Zhang Z., Lin X., Dong Y. (2014). Tissue culture-induced heritable genomic variation in rice, and their phenotypic implications. PLoS ONE.

[4] Azizi P., Hanafi M.M., Sahebi M., Harikrishna J.A., Taheri S., Yassoralipour A., Nasehi A. (2020). Epigenetic changes and their relationship to somaclonal variation: A need to monitor the micropropagation of plantation crops. Funct. Plant Biol..

[5] Jiang C., Mithani A., Gan X., Belfield E.J., Klingler J.P., Zhu J.K., Ragoussis J., Mott R., Harberd N.P. (2011). Regenerant Arabidopsis lineages display a distinct genome-wide spectrum of mutations conferring variant phenotypes. Curr. Biol..

[6] Yadav C.B., Pandey G., Muthamilarasan M., Prasad M. (2018). Epigenetics and Epigenomics of Plants. Adv. Biochem. Eng. Biotechnol..

[7] Lee M., Phillips R.L. (1988). The Chromosomal Basis of Somaclonal Variation. Annu. Rev. Plant Physiol. Plant Mol. Biol..

[8] Ong-Abdullah M., Ordway J.M., Jiang N., Ooi S.E., Kok S.Y., Sarpan N., Azimi N., Hashim A.T., Ishak Z.Z., Rosli S.K. (2015). Loss of Karma transposon methylation underlies the mantled somaclonal variant of oil palm. Nature.

[9] Miyao A., Nakagome M., Ohnuma T., Yamagata H., Kanamori H., Katayose Y., Takahashi A., Matsumoto T., Hirochika H. (2012). Molecular Spectrum of Somaclonal Variation in Regenerated Rice Revealed by Whole-Genome Sequencing. Plant Cell Physiol..

[10] Bednarek P.T., Orłowska R., Koebner R.M.D., Zimny J. (2007). Quantification of the tissue-culture induced variation in barley (*Hordeum vulgare* L.). BMC Plant Biol..

[11] Linacero R., Rueda J., Esquivel E., Bellido A., Domingo A., Vázquez A.M. (2011). Genetic and epigenetic relationship in rye, *Secale cereale* L., somaclonal variation within somatic embryo-derived plants. Vitr. Cell Dev. Biol. Plant.

[12] Krishna H., Alizadeh M., Singh D., Singh U., Chauhan N., Eftekhari M., Sadh R.K. (2016). Somaclonal variations and their applications in horticultural crops improvement. 3 Biotech.

[13] Smulders M.J.M., de Klerk G.J. (2011). Epigenetics in plant tissue culture. Plant Growth Regul..

[14] Evans D.A. (1989). Somaclonal variation—Genetic basis and breeding applications. Trends Genet..

[15] González G.A., Pacheco M.G., Oneto C.D., Etchart V.J., Kandus M.V., Salerno J.C., Eyherabide G., Presello D., Lewi D.M. (2012). Somatic embryogenesis and plant regeneration capacity in Argentinean maize (*Zea mays* L.) inbred lines. Electron. J. Biotechnol..

[16] Meng L., Zhang S., Lemaux P.G. (2010). Toward molecular understanding of in vitro and in planta shoot organogenesis. CRC Crit. Rev. Plant Sci..

[17] Shahid S., Axtell M.J. (2014). Identification and annotation of small RNA genes using ShortStack. Methods.

[18] Axtell M.J., Meyers B.C. (2018). Revisiting criteria for plant microRNA annotation in the era of big data. Plant Cell.

[19] Vashisht D., Nodine M.D. (2014). MicroRNA functions in plant embryos. Biochem. Soc. Trans..

[20] Singh A., Gautam V., Singh S., Sarkar Das S., Verma S., Mishra V., Mukherjee S., Sarkar A.K. (2018). Plant small RNAs: Advancement in the understanding of biogenesis and role in plant development. Planta.

[21] Zhang M., Dong Y., Nie L., Lu M., Fu C., Yu L. (2015). High-throughput sequencing reveals miRNA effects on the primary and secondary production properties in long-term subcultured Taxus cells. Front. Plant Sci..

[22] Chen Y., Zhang M., Jin X., Tao H., Wang Y., Peng B., Fu C., Yu L. (2020). Transcriptional reprogramming strategies and miRNA-mediated regulation networks of Taxus media induced into callus cells from tissues. BMC Genom..

[23] Rodriguez-Enriquez J., Dickinson H.G., Grant-Downton R.T. (2011). MicroRNA misregulation: An overlooked factor generating somaclonal variation?. Trends Plant Sci..

[24] Li H., Zhao X., Dai H., Wu W., Mao W., Zhang Z. (2012). Tissue culture responsive microRNAs in strawberry. Plant Mol. Biol. Rep..

[25] Morin R.D., Aksay G., Dolgosheina E., Ebhardt H.A., Magrini V., Mardis E.R., Sahinalp S.C., Unrau P.J. (2008). Comparative analysis of the small RNA transcriptomes of *Pinus contorta* and *Oryza Sativa*. Genome Res..

[26] Ghani M.A., Li J., Rao L., Raza M.A., Cao L., Yu N., Zou X., Chen L. (2014). The role of small RNAs in wide hybridisation and allopolyploidisation between *Brassica rapa* and *Brassica nigra*. BMC Plant Biol..

[27] Ha M., Pang M., Agarwal V., Chen Z.J. (2008). Interspecies regulation of microRNAs and their targets. Biochim. Biophys. Acta.

[28] Moturu T.R., Sinha S., Salava H., Thula S., Nodzyński T., Vařeková R.S., Friml J., Simon S. (2020). Molecular Evolution and Diversification of Proteins Involved in miRNA Maturation Pathway. Plants.

[29] Peng C., Chen X., Wang X., Xu X., Wei W., Wang C., Xu J. (2018). Comparative analysis of miRNA expression profiles in transgenic and non-transgenic rice using miRNA-Seq. Sci. Rep..

[30] Szwacka M., Pawełkowicz M., Skarzyńska A., Osipowski P., Wojcieszek M., Przybecki Z., Pląder W. (2018). Biological significance, computational analysis, and applications of plant microRNAs. Acta Physiol. Plant..

[31] Sun C., Zhao Q., Liu D.D., You C.X., Hao Y.J. (2013). Ectopic expression of the apple Md-miRNA156h gene regulates flower and fruit development in Arabidopsis. Plant Cell Tissue Org..

[32] Tang F., Wei H.R., Zhao S.T., Wang L.J., Zheng H.Q., Lu M.Z. (2016). Identification of microRNAs involved in regeneration of the secondary vascular system in Populus tomentosa Carr. Front. Plant Sci..

[33] Zhao Z., Xue Y.D., Yang H.L., Li H.M., Sun G.Y., Zhao X.F., Ding D., Tang J.H. (2016). Genome-wide identification of miRNAs and their targets involved in the developing internodes under maize ears by responding to hormone signaling. PLoS ONE.

[34] Ding D., Zhang L.F., Wang H., Liu Z.J., Zhang Z.X., Zheng Y.L. (2009). Differential expression of miRNAs in response to salt stress in maize roots. Ann. Bot..

[35] Kantar M., Lucas S.J., Budak H. (2011). miRNA expression patterns of Triticum dicoccoides in response to shock drought stress. Planta.

[36] Martinez G., Forment J., Llave C., Pallas V., Gomez G. (2011). High-throughput sequencing, characterization and detection of new and conserved cucumber miRNAs. PLoS ONE.

[37] Mao W., Li Z., Xia X., Li Y., Yu J. (2012). A combined approach of high-throughput sequencing and degradome analysis reveals tissue specific expression of microRNAs and their targets in cucumber. PLoS ONE.

[38] Ling J., Luo Z., Liu F., Mao Z., Yang Y., Xie B. (2017). Genome-wide analysis of microRNA targeting impacted by SNPs in cucumber genome. BMC Genom..

[39] Sun Y., Luo W., Chang H., Li Z., Zhou J., Li X., Zheng J., Hao M. (2019). Identification of miRNAs and Their Target Genes Involved in Cucumber Fruit Expansion Using Small RNA and Degradome Sequencing. Biomolecules.

[40] Skarzyńska A., Pawełkowicz M., Pląder W. (2020). Genome-wide discovery of DNA variants in cucumber somaclonal lines. Gene.

[41] Pawełkowicz M.E., Skarzyńska A., Mróz T., Bystrzycki E., Pląder W. (2021). Molecular insight into somaclonal variation phenomena from transcriptome profiling of cucumber (*Cucumis sativus* L.) lines. Plant Cell Tissue Organ Cult..

[42] Salmena L., Poliseno L., Tay Y., Kats L., Pandolfi P.P. (2011). A ceRNA hypothesis: The Rosetta Stone of a hidden RNA language?. Cell.

[43] Yang X., Wang L., Yuan D., Lindsey K., Zhang X. (2013). Small RNA and degradome sequencing reveal complex miRNA regulation during cotton somatic embryogenesis. J. Exp. Bot..

[44] Wang Q., Zhang B. (2015). MicroRNAs in cotton: An open world needs more exploration. Planta.

[45] Barabási A.L. (2012). The network takeover. Nat. Phys..

[46] Boccaletti S., Latora V., Moreno Y., Chavez M., Hwang D.U. (2006). Complex networks: Structure and dynamics. Phys. Rep..

[47] Newman M.E.J. (2010). Networks: An Introduction.

[48] Smith K.M. (2019). On neighbourhood degree sequences of complex networks. Sci. Rep..

[49] Shepherd V.A. (2006). The cytomatrix as a cooperative system of macromolecular and water networks. Curr. Top. Dev. Biol..

[50] Chen Y., Weckwerth W. (2020). Mass spectrometry untangles plant membrane protein signaling networks. Trends Plant Sci..

[51] Salih H., Gong W., He S., Sun G., Sun J., Du X. (2016). Genome-wide characterization and expression analysis of MYB transcription factors in *Gossypium hirsutum*. BMC Genet..

[52] Orenstein Y., Shamir R. (2017). Modeling protein–DNA binding via high-throughput in vitro technologies. Brief. Funct. Genom..

[53] Boeva V. (2016). Analysis of genomic sequence motifs for deciphering transcription factor binding and transcriptional regulation in eukaryotic cells. Front. Genet..

[54] Samad A.F.A., Sajad M., Nazaruddin N., Fauzi I.A., Murad A.M.A., Zainal Z., Ismail I. (2017). MicroRNA and Transcription Factor: Key Players in Plant Regulatory Network. Front. Plant Sci..

[55] Jones-Rhoades M.W., Bartel D.P. (2004). Computational identification of plant microRNAs and their targets, including a stress-induced miRNA. Mol. Cell.

[56] Xu G., Ma H., Nei M., Kong H. (2009). Evolution of F-box genes in plants: Different modes of sequence divergence and their relationships with functional diversification. Proc. Natl. Acad. Sci. USA.

[57] Zhang T., Lv W., Zhang H., Ma L., Li P., Ge L., Li G. (2018). Genome-wide analysis of the basic Helix-Loop-Helix (bHLH) transcription factor family in maize. BMC Plant Biol..

[58] Garcia-Mas J., Benjak A., Sanseverino W., Bourgeois M., Mir G., Gonzalez V.M., Henaff E., Camara F., Cozzuto L., Lowy E. (2012). Lorent The genome of melon (*Cucumis melo* L.). Proc. Natl. Acad. Sci. USA.

[59] Xu M., Hu T., Zhao J., Park M.Y., Earley K.W., Wu G., Yang L., Poethig R.S. (2016). Developmental Functions of miR156-Regulated SQUAMOSA PROMOTER BINDING PROTEIN-LIKE (SPL) Genes in *Arabidopsis thaliana*. PLoS Genet..

[60] Long J.M., Liu C.Y., Feng M.Q., Liu Y., Wu X.M., Guo W.W. (2018). MiR156-SPL modules regulate induction of somatic embryogenesis in citrus callus. J. Exp. Bot..

[61] Rhoades M.W., Reinhart B.J., Lim L.P., Burge C.B., Bartel B., Bartel D.P. (2002). Prediction of plant microRNA targets. Cell.

[62] Gonzalez-Ibeas D., Blanca J., Donaire L., Saladié M., Mascarell-Creus A., Cano-Delgado A., Garcia-Mas J., Llave C., Aranda M.A. (2011). Analysis of the melon (*Cucumis melo*) small RNAome by high-throughput pyrosequencing. BMC Genom..

[63] Laufs P. (2004). MicroRNA regulation of the CUC genes is required for boundary size control in Arabidopsis meristems. Development.

[64] Ikeda M., Ohme-Takagi M. (2014). TCPs, WUSs, and WINDs: Families of transcription factors that regulate shoot meristem formation, stem cell maintenance, and somatic cell differentiation. Front. Plant Sci..

[65] Rodriguez R.E., Ercoli M.F., Debernardi J.M., Breakfield N.W., Mecchia M.A., Sabatini M., Cools T., De Veylder L.L., Benfey P.N., Palatnik J.F. (2015). MicroRNA miR396 regulates the switch between stem cells and transit-amplifying cells in Arabidopsis roots. Plant Cell.

[66] Budak H., Kantar M., Bulut R., Akpinar B.A. (2015). Stress responsive miRNAs and isomiRs in cereals. Plant Sci..

[67] Miguel C., Marum L. (2011). An epigenetic view of plant cells cultured in vitro: Somaclonal variation and beyond. J. Exp. Bot..

[68] Ranghoo-Sanmukhiya V.M., Siddique I. (2021). Somaclonal variation and methods used for its detection. Propagation and Genetic Manipulation of Plants.

[69] Malepszy S., Burza W., Smiech M. (1996). Characterization of a cucumber (*Cucumis sativus* L.) somaclonal variant with paternal inheritance. J. Appl. Genet..

[70] Bartoszewski G., Malepszy S., Havey M.J. (2004). Mosaic (MSC) cucumbers regenerated from independent cell cultures possess different mitochondrial rearrangements. Curr. Genet..

[71] Lilly J.W., Bartoszewski G., Malepszy S., Havey M.J. (2001). A major deletion in the cucumber mitochondrial genome sorts with the MSC phenotype. Curr. Genet..

[72] Plader W., Malepszy S., Burza W., Rusinowski Z. (1998). The relationship between the regeneration system and genetic variability in the cucumber (*Cucumis sativus* L.). Euphytica.

[73] Ładyżyński M., Burza W., Malepszy S. (2002). Relationship between somaclonal variation and type of culture in cucumber. Euphytica.

[74] FastQC Software. https://www.bioinformatics.babraham.ac.uk/projects/fastqc/.

[75] Altschul S.F., Gish W., Miller W., Myers E.W., Lipman D.J. (1990). Basic local alignment search tool. J. Mol. Biol..

[76] Kozomara A., Griffiths-Jones S. (2014). miRBase: Annotating high confidence microRNAs using deep sequencing data. Nucleic Acids Res..

[77] Love M.I., Huber W., Anders S. (2014). Moderated estimation of fold change and dispersion for RNAseq data with DESeq2. Genome Biol..

[78] psRNA Target Software. http://plantgrn.noble.org/psRNATarget/.

[79] Götz S., Garcia-Gomez J.M., Terol J., Williams T.D., Nagaraj S.H., Nueda M.J., Robles M., Talon M., Dopazo J., Conesa A. (2008). High-throughput functional annotation and data mining with the Blast2GO suite. Nucleic Acids Res..

[80] RNA Folder Software. http://www.ncrnalab.dk/#rnafolder/rnafolder.php.

[81] Zhang K., He S., Sui Y., Gao Q., Jia S., Lu X., Jia L. (2021). Genome-Wide Characterization of HSP90 Gene Family in Cucumber and Their Potential Roles in Response to Abiotic and Biotic Stresses. Front. Genet..

[82] Zhang X., Lai Y., Zhang W., Ahmad J., Qiu Y., Zhang X., Duan M., Liu T., Song J., Wang S. (2018). MicroRNAs and their targets in cucumber shoot apices in response to temperature and photoperiod. BMC Genom..

[83] Folkes L., Moxon S., Woolfenden H.C., Stocks M.B., Szittya G., Dalmay T., Moulton V. (2012). PAREsnip: A tool for rapid genome-wide discovery of small RNA/target interactions evidenced through degradome sequencing. Nucleic Acids Res..

[84] Osipowski P., Pawełkowicz M., Wojcieszek M., Skarzyńska A., Przybecki Z., Pląder W. (2020). A high-quality cucumber genome assembly enhances computational comparative genomics. Mol. Genet. Genom..

[85] Szklarczyk D., Morris J.H., Cook H., Kuhn M., Wyder S., Simonovic M., Santos A., Doncheva N.T., Roth A., Bork P. (2016). The STRING database in 2017: Quality-controlled protein–protein association networks, made broadly accessible. Nucleic Acids Res..

[86] Cytoscape Software. https://cytoscape.org/.

[87] Androvic P., Valihrach L., Elling J., Sjoback R., Kubista M. (2017). Two-tailed RT-qPCR: A novel method for highly accurate miRNA quantification. Nucleic Acids Res..

