# Supplementary material for: miRNA Profiling and Its Role in Multi-Omics Regulatory Networks Connected with Somaclonal Variation in Cucumber (Cucumis sativus L.)
Source: Int J Mol Sci. 2022 Apr 13;23(8):4317. doi: 10.3390/ijms23084317 (PMC9031375; doi:10.3390/ijms23084317)

pre-cst-novel-miR9
ΔG=-56.03 kcal/mol

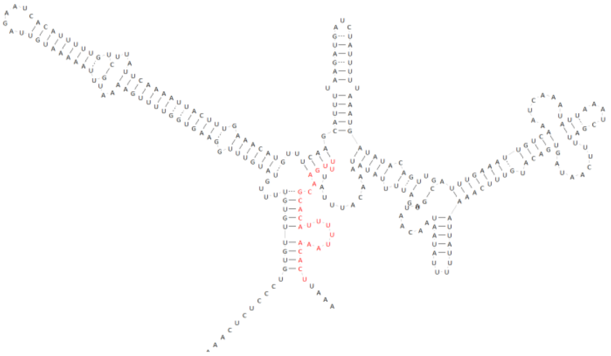

pre-cst-novel-miR26
ΔG=-111.80 kcal/mol

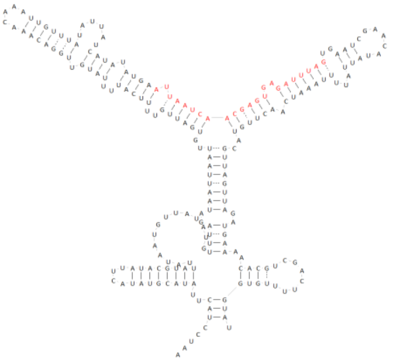

pre-cst-novel-miR39
ΔG=-94.10 kcal/mol

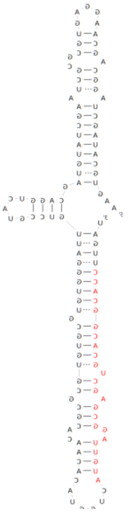

pre-cst-novel-miR94
ΔG=-104.05 kcal/mol

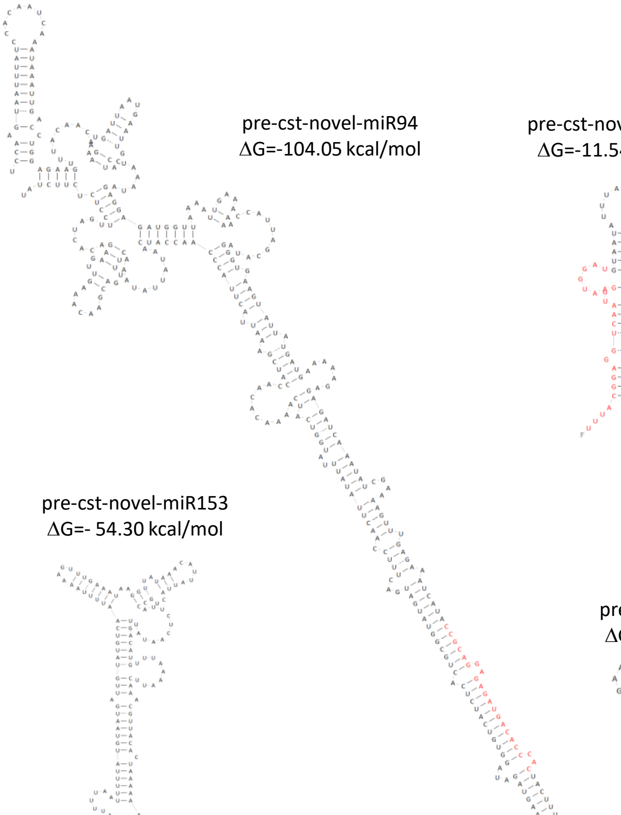

pre-cst-novel-miR104
ΔG=-11.54 kcal/mol

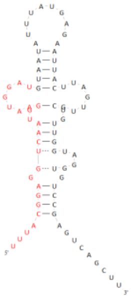

pre-cst-novel-miR114
ΔG= -18.81 kcal/mol

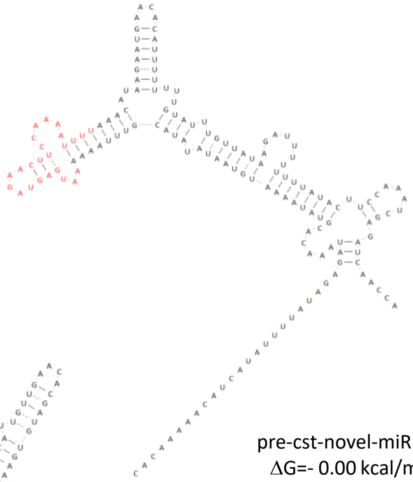

pre-cst-novel-miR153
ΔG=- 54.30 kcal/mol

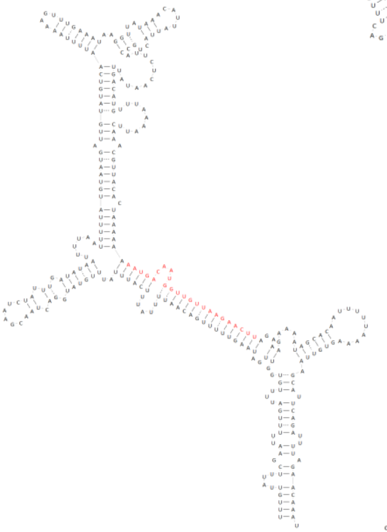

pre-cst-novel-miR138
ΔG=- 76.62 kcal/mol

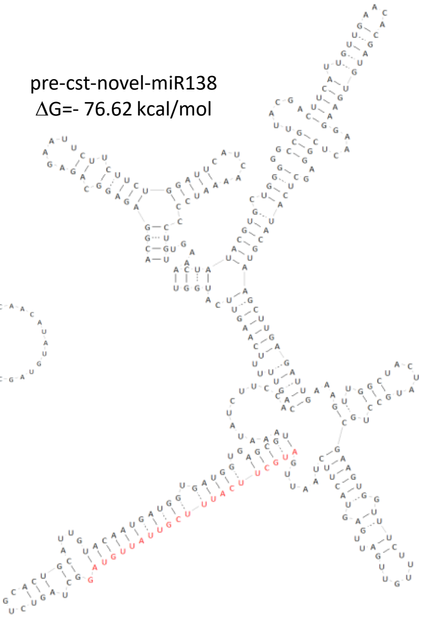

pre-cst-novel-miR156
ΔG=- 0.00 kcal/mol

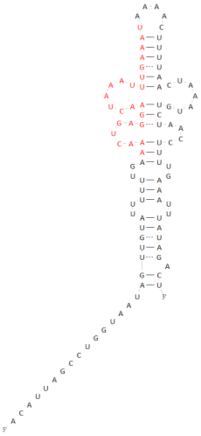

pre-cst-novel-miR120
ΔG=- 103.50 kcal/mol

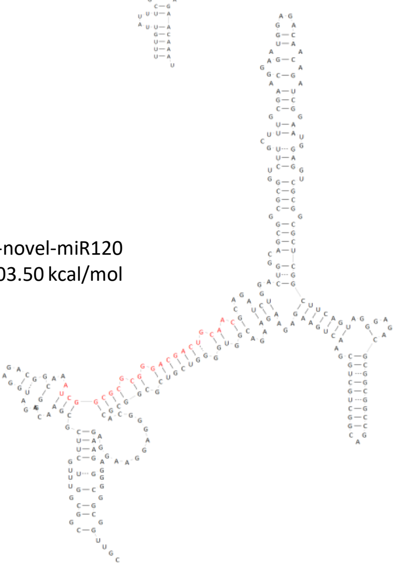

pre-cst-novel-miR167
ΔG=- 86.43 kcal/mol

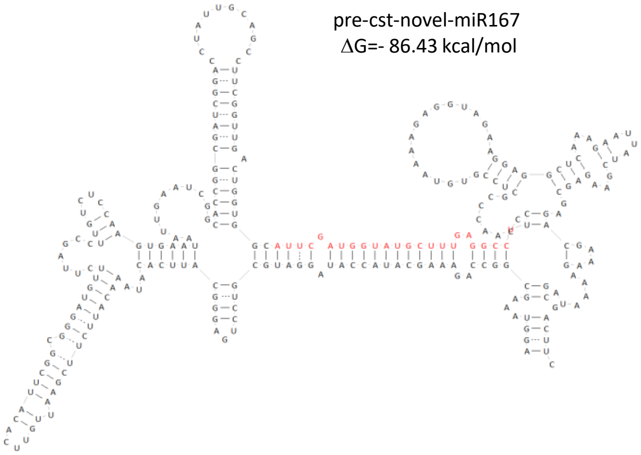

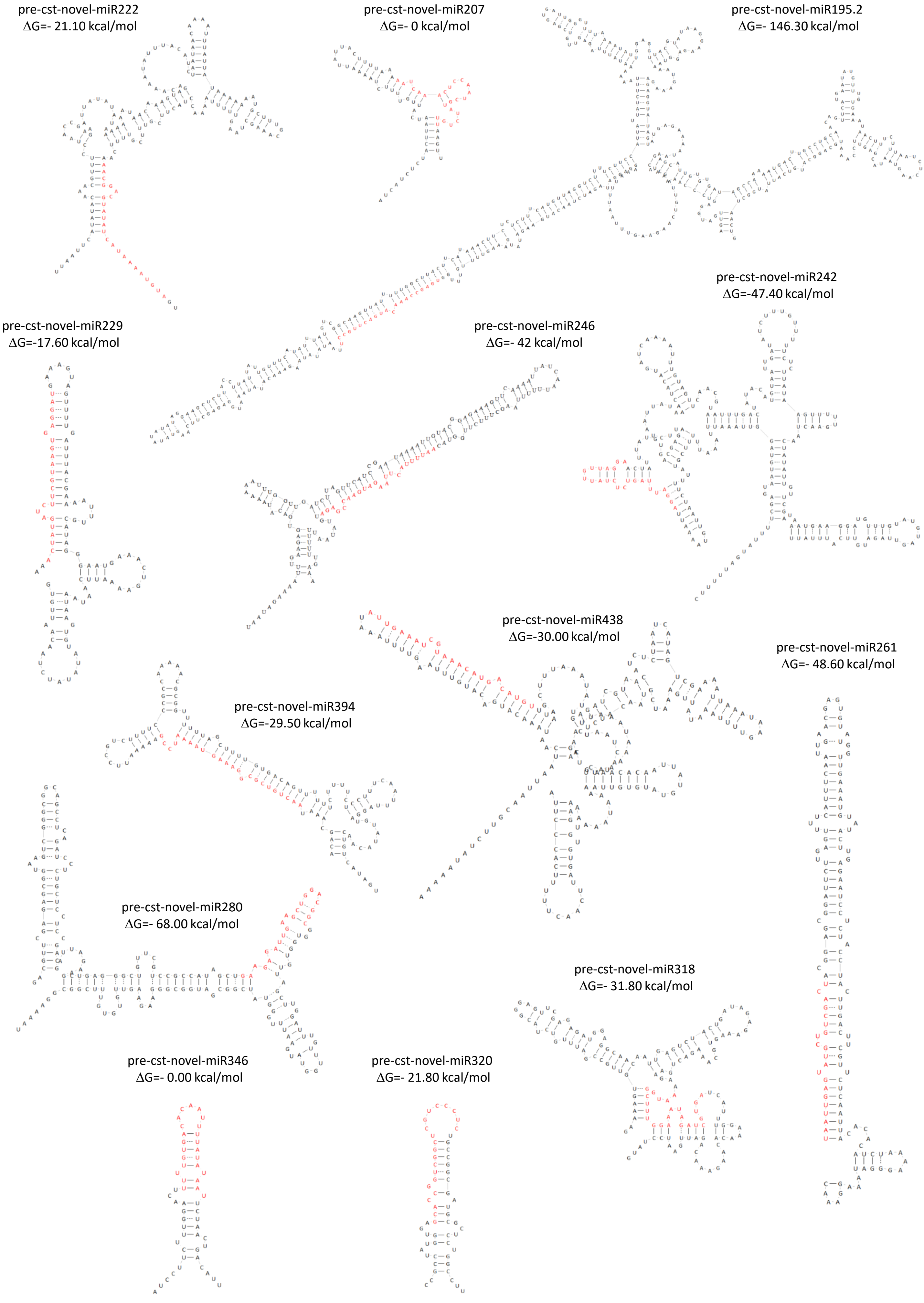
pre-cst-novel-miR222
ΔG=- 21.10 kcal/mol
pre-cst-novel-miR207
ΔG=- 0 kcal/mol
pre-cst-novel-miR195.2
ΔG=- 146.30 kcal/mol
pre-cst-novel-miR242
ΔG=-47.40 kcal/mol
pre-cst-novel-miR229
ΔG=-17.60 kcal/mol
pre-cst-novel-miR246
ΔG=- 42 kcal/mol
pre-cst-novel-miR438
ΔG=-30.00 kcal/mol
pre-cst-novel-miR261
ΔG=- 48.60 kcal/mol
pre-cst-novel-miR394
ΔG=-29.50 kcal/mol
pre-cst-novel-miR280
ΔG=- 68.00 kcal/mol
pre-cst-novel-miR318
ΔG=- 31.80 kcal/mol
pre-cst-novel-miR346
ΔG=- 0.00 kcal/mol
pre-cst-novel-miR320
ΔG=- 21.80 kcal/mol

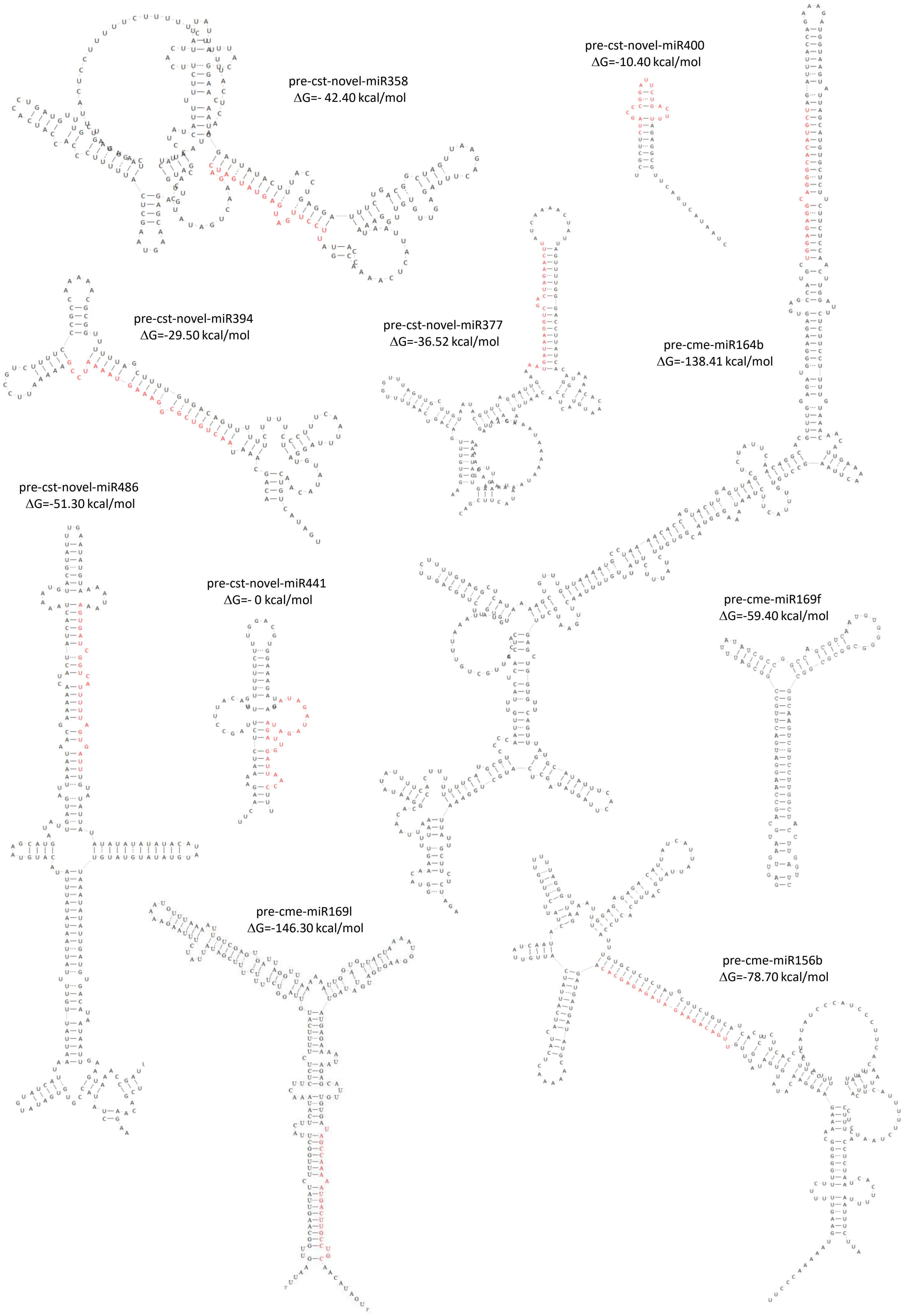
pre-cst-novel-miR358
ΔG=- 42.40 kcal/mol
pre-cst-novel-miR400
ΔG=-10.40 kcal/mol
pre-cst-novel-miR394
ΔG=-29.50 kcal/mol
pre-cst-novel-miR377
ΔG=-36.52 kcal/mol
pre-cme-miR164b
ΔG=-138.41 kcal/mol
pre-cst-novel-miR486
ΔG=-51.30 kcal/mol
pre-cst-novel-miR441
ΔG=- 0 kcal/mol
pre-cme-miR169f
ΔG=-59.40 kcal/mol
pre-cme-miR169l
ΔG=-146.30 kcal/mol
pre-cme-miR156b
ΔG=-78.70 kcal/mol

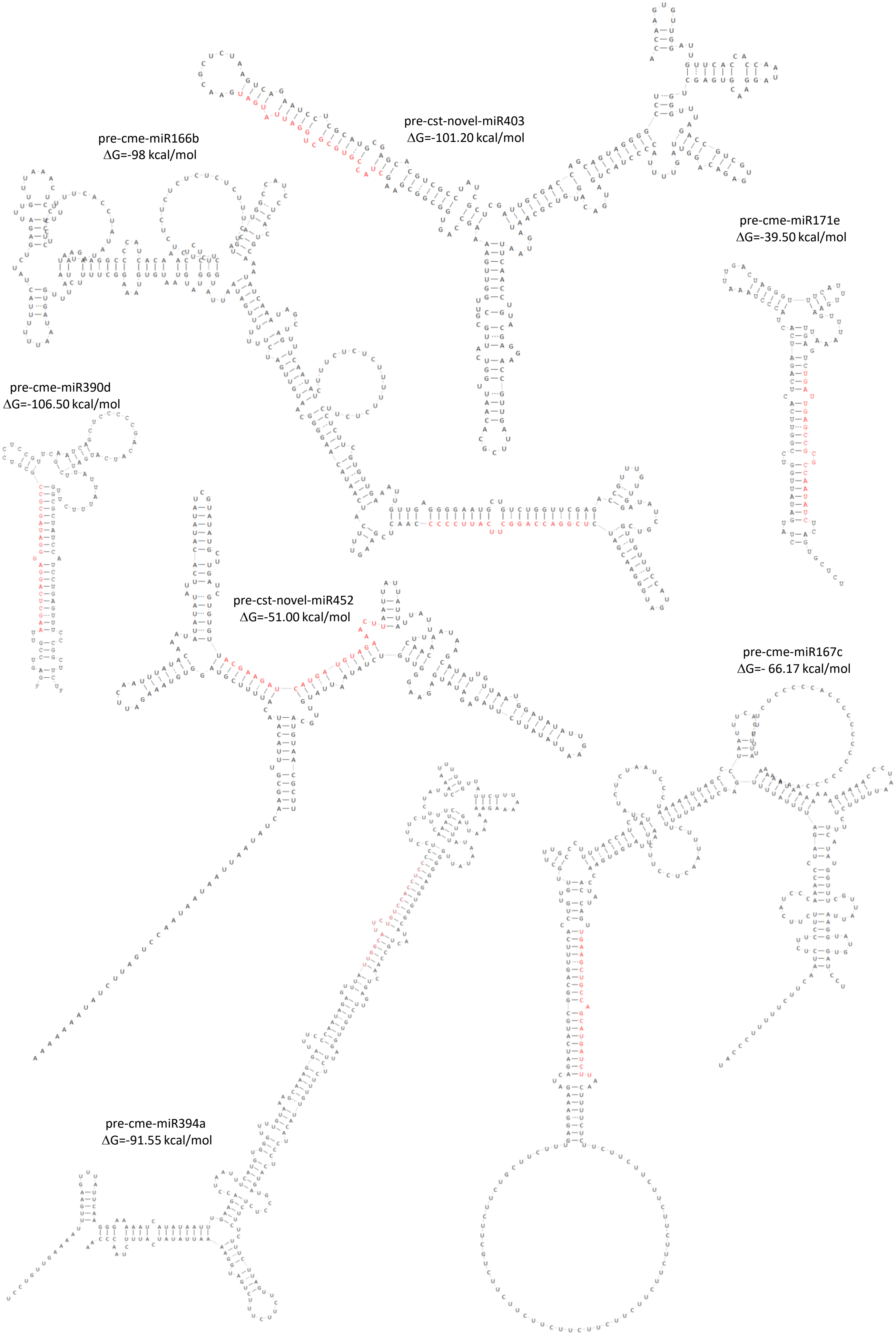
pre-cme-miR166b
ΔG=-98 kcal/mol
pre-cst-novel-miR403
ΔG=-101.20 kcal/mol
pre-cme-miR171e
ΔG=-39.50 kcal/mol
pre-cme-miR390d
ΔG=-106.50 kcal/mol
pre-cst-novel-miR452
ΔG=-51.00 kcal/mol
pre-cme-miR167c
ΔG=- 66.17 kcal/mol
pre-cme-miR394a
ΔG=-91.55 kcal/mol

pre-cme-miR396e
ΔG=-70.80 kcal/mol

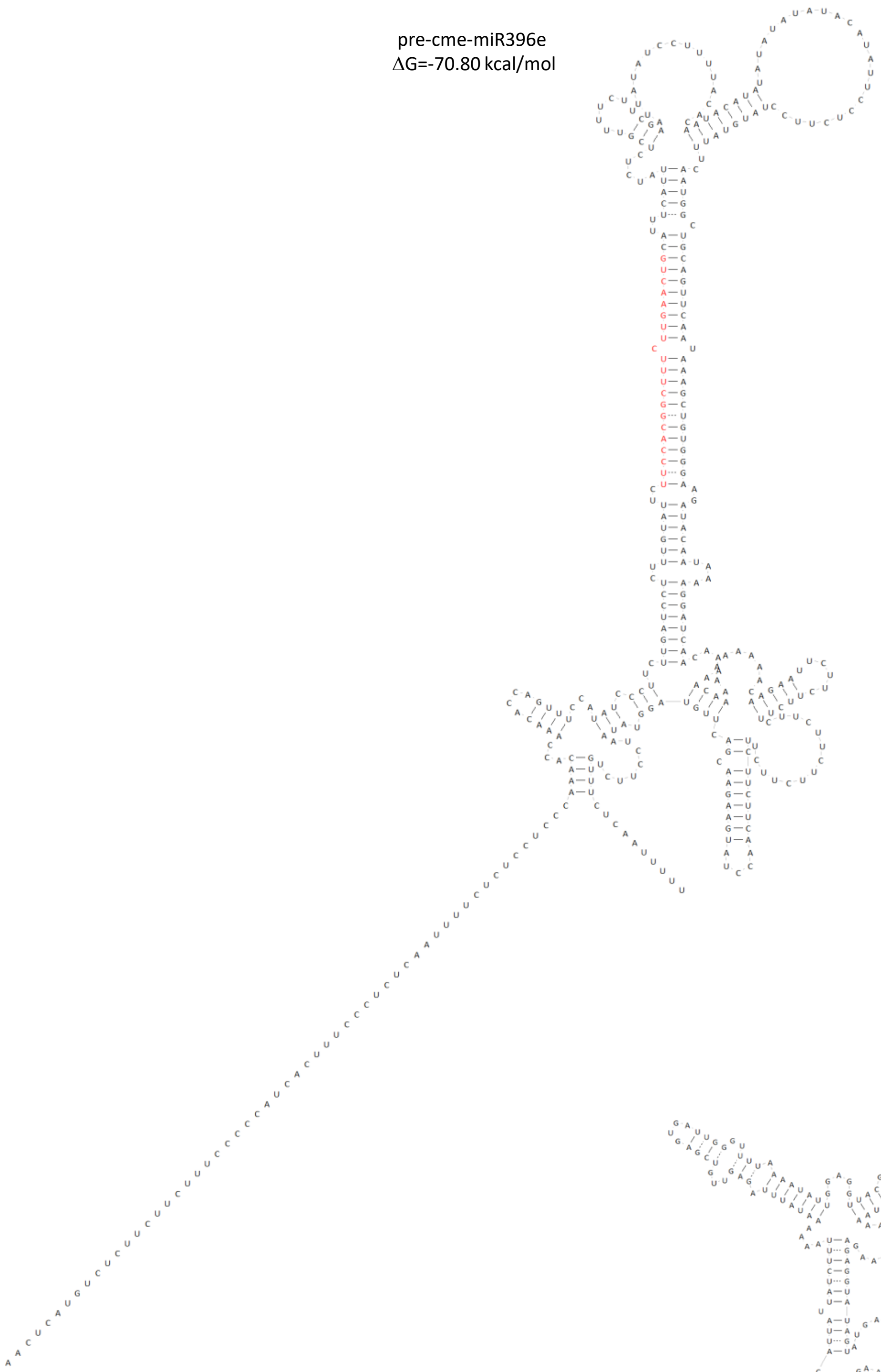

pre-cst-novel-miR196.2
ΔG=-145.60 kcal/mol

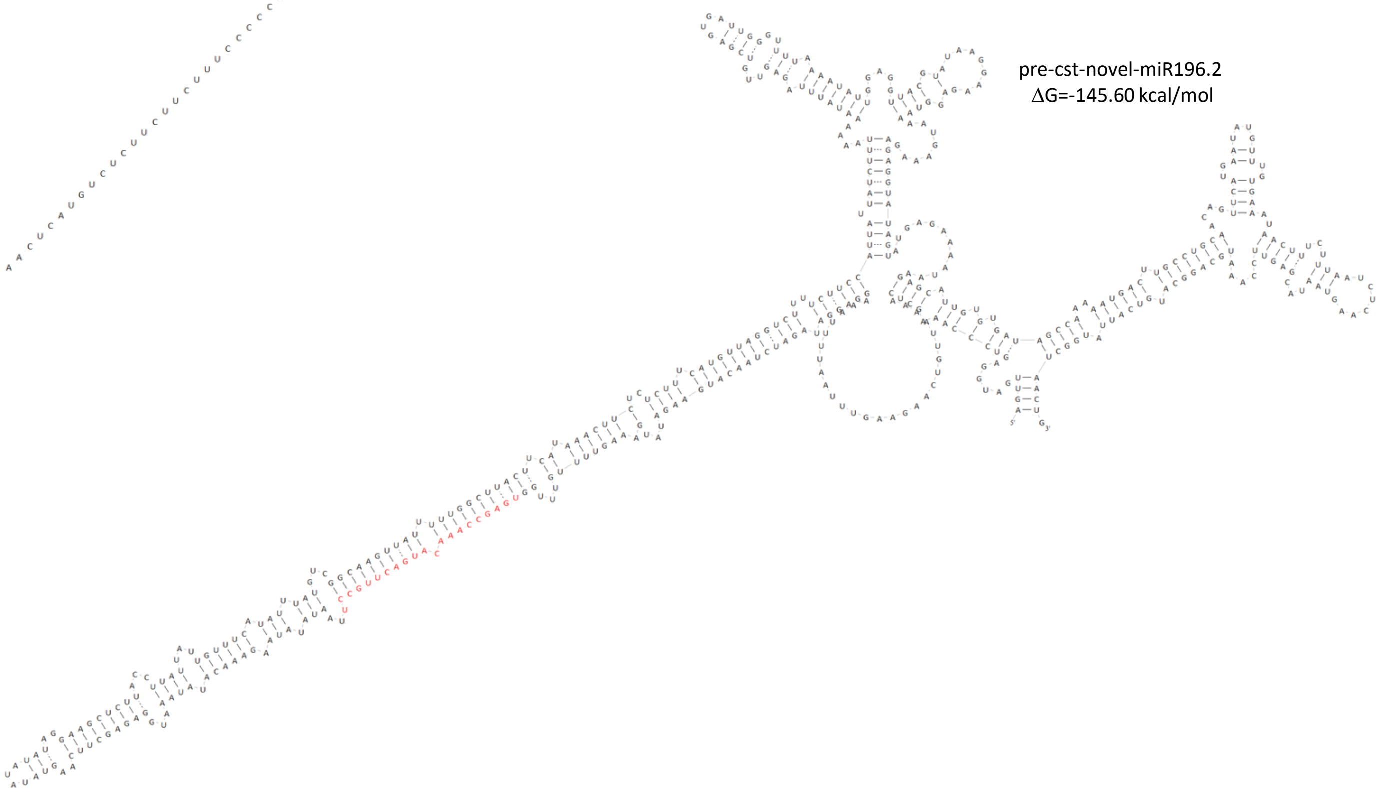

Supplement: Supplementary file 1 [file ijms-23-04317-s001.zip › Figure S1.pdf]
