# Supplementary figures and images for: miRNA Profiling and Its Role in Multi-Omics Regulatory Networks Connected with Somaclonal Variation in Cucumber (Cucumis sativus L.)
Source: Int J Mol Sci. 2022 Apr 13;23(8):4317. doi: 10.3390/ijms23084317 (PMC9031375; doi:10.3390/ijms23084317)

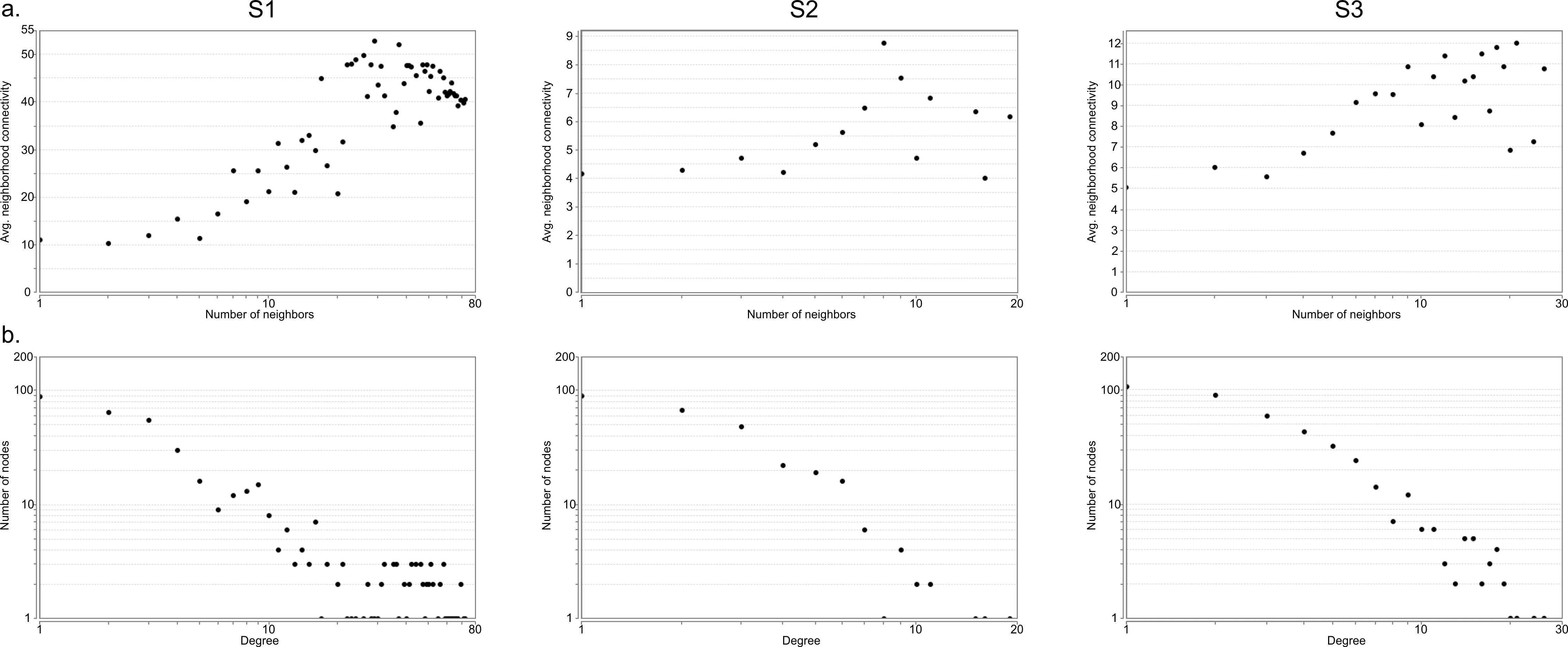

Supplement: Supplementary file 1 [file ijms-23-04317-s001.zip › Figure S2.png]
